# Impacts of amino acid supplementation on renal function and nutritional parameters in patients with renal insufficiency: bibliometric analysis and meta-analysis

**DOI:** 10.3389/fnut.2025.1594507

**Published:** 2025-06-13

**Authors:** Xiaoxia Liu, Qiufu Li, Liyuan Zhang, Ying He, Sitao Tan, Xiaoyu Chen, Kefeng Li

**Affiliations:** 1 Center for Artificial Intelligence Driven Drug Discovery, Faculty of Applied Sciences, Macao Polytechnic University, Macao, China; 2 Department of Pharmacy, The People's Hospital of Guangxi Zhuang Autonomous Region, Guangxi Academy of Medical Sciences, Nanning, China; 3 Phase 1 Clinical Trial Laboratory, The People's Hospital of Guangxi Zhuang Autonomous Region, Guangxi Academy of Medical Sciences, Nanning, China

**Keywords:** renal insufficiency, amino acids, meta-analysis, bibliometrics, renal function, nutritional indicators

## Abstract

**Objective:**

The aim of this study was to summarize the effects of amino acids (AA) on renal function and nutritional indices in patients with renal insufficiency (RI) after treatment and to analyze the development trend in this field.

**Methods:**

The bibliometric evaluation of scholarly contributions in this field was conducted using the Web of Science database, with data analyzed via Bibliometrix and VOSviewer software. The randomized controlled trials (RCTs) published before January 13, 2025, were systematically retrieved from Embase, PubMed, and the Cochrane Library and meta-analyses were performed using Review Manager 5.4 software.

**Results:**

Key areas of focus included oxidative stress, chronic renal failure, hemodialysis, inflammation, chronic kidney disease, risk, plasma, progression, L-arginine, disease, and renal failure. Nine RCTs involving 407 participants were included, AA administration demonstrated significant effects compared to placebo: Increased blood urea nitrogen (MD: 4.21, 95% CI: 1.08 to 7.35, *p* = 0.008), elevated renal plasma flow (MD: 30.78, 95% CI: 15.36 to 46.21, *p* < 0.0001), and reduced uric acid levels (MD: −0.47, 95% CI: −0.89 to −0.06, *p* = 0.02).

**Conclusion:**

These findings suggest that AA supplementation may partially improve renal function in RI patients. The progression and possible mechanisms of chronic kidney disease, as well as the search for new biomarkers, will be the trend of research and development in this field.

## 1 Introduction

Renal insufficiency (RI) is a clinical syndrome characterized by impaired kidney metabolic function resulting from diverse etiologies, leading to disruptions in the body’s metabolites, fluid balance, electrolytes, and acid–base homeostasis (1). RI is categorized into chronic renal insufficiency and acute renal insufficiency (2). However, inconsistent terminology in renal function and disease classification has prompted standardized nomenclature: chronic renal insufficiency is now termed chronic kidney disease (CKD) per Kidney Disease Improving Global Outcomes (KDIGO) conference consensus (3, 4), while acute renal insufficiency is defined as acute kidney injury (5–7). CKD encompasses pathologies such as glomerulonephritis, diabetic nephropathy, and end-stage renal disease (8, 9), and is staged 1–5 based on severity, progressing from mild functional impairment to renal failure requiring dialysis or transplantation. Projections indicate that by 2040, kidney disease will rank as the fifth leading cause of reduced life expectancy globally (4, 8).

Amino acids (AA) and their derivatives are ubiquitously utilized as dietary supplements in clinical and nutritional contexts, exerting profound influences on renal physiology and homeostasis. RI is associated with dysregulated serum AA concentrations, though evidence suggests moderate dietary AA intake may confer therapeutic benefits (1). Studies indicate that AA infusion protects renal function through mechanisms such as replenishing depleted AA, enhancing renal plasma flow (RPF), and improving estimated glomerular filtration rate (10–12). The kidneys also contribute significantly to protein metabolism: in healthy individuals, 97–98% of filtered AA and peptides undergo tubular reabsorption. In CKD patients, however, inflammatory states and metabolic acidosis disrupt AA and protein metabolism. Progressive CKD further alters filtration and reabsorption, exacerbating AA wasting and proteinuria (13, 14).

AA and their derivatives, as fundamental components of proteins and peptides, serve as critical dietary supplements with profound implications for renal health (15). AA exhibits multifaceted roles in nutrition, therapy, and medicine, frequently acting as a primary active ingredient in nutritional formulations (16). Biomarkers, such as albumin (ALB), total protein (TP), and transferrin (TRF), are indispensable for evaluating nutritional status in patients with RI, while also serving as predictors of disease progression and clinical outcomes (17). Despite their therapeutic potential, excessive intake of most AA supplements, particularly amino acids like glutamine and arginine, which are essential for tumor cell proliferation, may induce adverse effects (18). In RI patients, prolonged high-dose AA administration can disrupt amino acid homeostasis, leading to nitrogen accumulation and exacerbating renal damage (19).

Preclinical studies have demonstrated that AA supplementation improves glomerular filtration rate (GFR) in RI models, yet clinical trials report inconsistent outcomes. This discrepancy is compounded by substantial heterogeneity in study design, including variations in AA formulations, intervention durations, and patient baseline characteristics (20, 21). Notably, while AA metabolism is intricately linked to nutritional status, current research has underemphasized key nutritional parameters—such as ALB, TP, TRF, and body mass index (BMI)—which are central to assessing both nutritional adequacy and renal disease trajectories (17). Existing literature further highlights conflicting evidence regarding AA’s effects on these markers: divergent results have been reported for ALB, TP, and TRF in RI populations undergoing AA interventions. Although animal studies suggest renoprotective and nutritional benefits, the clinical evidence base remains fragmented, characterized by a lack of systematic quantitative synthesis, especially of high-quality randomized controlled trials (RCTs), hindering clarity on AA’s clinical applicability and optimal dosing strategies in RI (11, 22, 23). To address this gap, we conducted a bibliometric analysis to map research trends and a meta-analysis of RCTs to (1) evaluate AA effects on renal function: blood urea nitrogen (BUN), RPF, GFR, uric acid (UA), and nutritional parameters; (2) identify intervention modifiers through subgroup analyses; and (3) delineate unresolved evidence gaps for future inquiry. This integrated approach aims to quantify the clinical benefits of AA in renal protection and nutritional efficacy, providing robust evidence to inform clinical practice.

## 2 Materials and methods

### 2.1 Bibliometric analysis

Guided by PubMed Medical Subject Headings (MeSH) terminology and the KDIGO conference consensus statement, we systematically retrieved articles published between 1 January 2000 and 31 December 2024 from the Web of Science database on 1 January 2025, using RI and AA as primary search keywords (7, 24). The complete search strategy is provided in Supplementary material S1, and detailed eligibility criteria are outlined in Supplementary material S2. For bibliometric evaluation, the following data were extracted from the selected literature: author information, journal affiliations, citation metrics, and institutional collaborations. Text analysis and data visualization were performed using R software (v4.4.1) and VOSviewer (v1.6.20) (25, 26).

### 2.2 Meta-analysis

**Figure 1:**
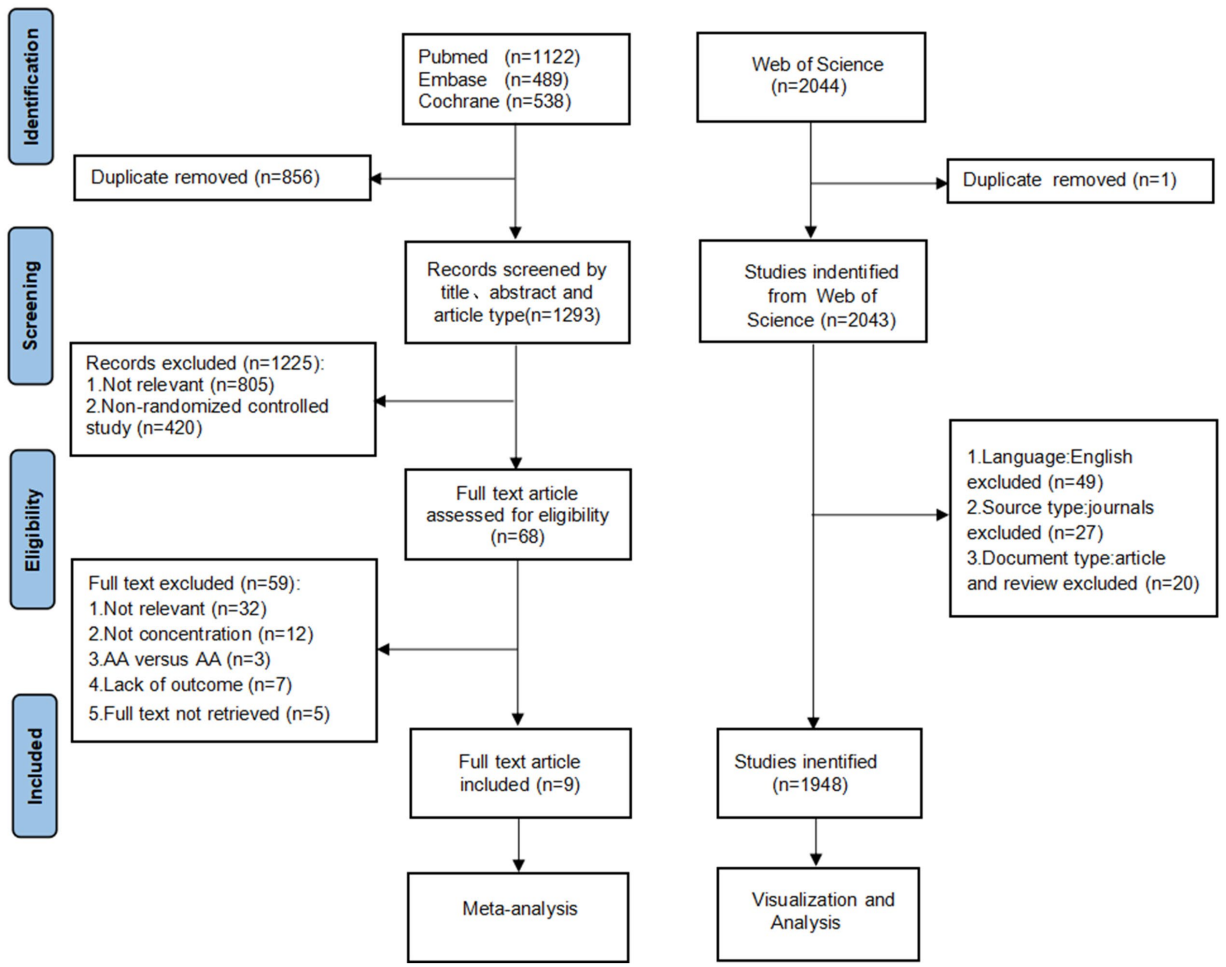
Flowchart of study selection for meta-analysis. AA, amino acids.

We registered the study on PROSPERO (CRD42024610476) in accordance with the Preferred Reporting Items for Systematic Reviews and Meta-Analyses (PRISMA) guidelines (27). A systematic search was conducted across Embase, PubMed, and the Cochrane Library to identify English-language articles examining the effects of AA supplementation on renal function and nutritional indicators in patients with RI. The search encompassed all available records from database inception until January 13, 2025. The search strategy utilized three core terms combined with the AND operator and MeSH subject headings: RCTs, AA, and RI (Supplementary material S1). The screening process is summarized in Figure 1. Two independent investigators (Liu and Li) screened titles, keywords, and abstracts against predefined inclusion and exclusion criteria (Supplementary material S2). Full-text review was performed to extract the following data: (1) study characteristics: lead author, year of publication, sample size; (2) study participant characteristics: age, gender, total number of participants, number of intervention groups, number of control groups, intervention and control measures, and duration of the intervention; (3) type of patients with RI; and (4) data on study outcomes: BUN, RPF, GFR, UA, ALB, TP, TRF, BMI. Data extraction was conducted independently by both investigators using standardized forms, with discrepancies resolved through consultation with a third researcher (Zhang). Of the initial 1,293 identified records, 1,225 were excluded during title/abstract screening due to duplication (856), irrelevance (805), or non-RCT design (420), leaving 68 articles for full-text evaluation. Full-text exclusion criteria included off-topic content, missing outcome data, control groups receiving AA supplementation, or inaccessible full-text materials.

Nine studies were ultimately included in the meta-analysis. For studies reporting standard errors instead of standard deviation (SD), conversions were performed using the formula (28):


$$\mathrm{SD}=\mathrm{SE}\times \sqrt{n}$$

Statistical analyses were conducted using Review Manager 5.4 (The Cochrane Collaboration, Oxford, United Kingdom) to generate forest plots and subgroup analyses. Publication bias and sensitivity analyses were performed using Stata 18.0 (StataCorp, College Station, TX, United States) (29, 30). Heterogeneity across studies was evaluated using the I^2^ value, with thresholds defined as follows: I^2^ ≤ 25% (low heterogeneity), 26–50% (moderate heterogeneity), and >50% (high heterogeneity). Sensitivity analyses were performed in cases of I^2^ > 50% or *p* < 0.05, and studies were excluded on a case-by-case basis to determine their impact on the overall estimate. Data were synthesized using either a fixed-effects or random-effects meta-analysis model, selected based on the degree of heterogeneity. A random-effects model was applied for I^2^ > 50%, while a fixed-effects model was used for I^2^ ≤ 50%. Continuous outcome measures were reported as Mean Difference (MD) and 95% confidence intervals (95% CI). Statistical significance was defined as *p* < 0.05 for all analyses.

### 2.3 Inclusion and exclusion criteria

#### 2.3.1 Bibliometric inclusion and exclusion criteria

The included studies met the following criteria: (1) focus on patients with renal insufficiency undergoing interventions involving amino acids; (2) peer-reviewed articles and literature reviews; and (3) publications dated between January 1, 2000, and December 31, 2024.

Excluded studies encompassed (1) animal studies; (2) non-academic materials (e.g., conference proceedings, news reports, patents, calls for papers, newspaper abstracts); (3) non-English publications; (4) journals irrelevant to the topic; and (5) articles lacking accessible data.

#### 2.3.2 Meta-analysis inclusion and exclusion criteria

To ensure precision, search results underwent manual screening during the meta-analysis, with post-screening studies validated twice and independently.

Inclusion criteria: (1) Population: Adult participants (≥18 years) diagnosed with renal impairment; (2) Study design: RCTs; (3) Language: English-language publications; (4) Intervention: Use of L-amino acids (individual or combined formulations) in the treatment group, compared to a placebo control; and (5) Outcomes: At least one renal function marker (BUN, RPF, GFR, UA) or nutritional indicator (ALB, TP, TRF, BMI).

Exclusion criteria: (1) animal or pediatric (<18 years) studies; (2) non-RCT designs (e.g., observational studies, case reports); (3) interventions involving non-L-amino acids, structurally modified L-amino acids (e.g., glutamine, acetylcysteine), or protein/amino acid-based nutritional supplements in control groups; and (4) insufficient outcome data or unavailable full-text articles.

### 2.4 Quality assessment

The risk of bias for each included trial was independently evaluated by two investigators using the Cochrane Risk of Bias Tool for Randomized Controlled Trials RoB 2 (31). This assessment focused on five domains: (1) bias arising from the randomization process; (2) bias due to deviations from intended interventions; (3) bias due to missing outcome data; (4) bias in outcome measurement; and (5) bias in the selection of reported results. Study quality was categorized as follows: High-risk studies: Trials with two or more domains rated as high risk of bias; low-risk studies: 5 or more low-risk studies and no more than 1 high-risk study; Studies with some concerns: Trials that did not meet criteria for low-risk or high-risk classifications (32). Publication bias for primary outcomes was assessed using Stata 18.0 software to generate funnel plots or perform Egger’s regression test (33).

## 3 Results

### 3.1 Results of bibliometric analysis

#### 3.1.1 General description of retrieved publications

To address source heterogeneity and language variability, inclusion criteria were restricted to English-language research articles. Further exclusions were applied based on document and publication type (Figure 1). A total of 1948 English-language publications on AA and RI patients’ renal function and nutrition were analyzed. Among these, original research articles predominated (1,530 articles, 75.54%), followed by reviews (418 articles, 21.46%).

#### 3.1.2 Trends in publication growth

Supplementary Figure S1 illustrates annual and cumulative publication trends from 2000 to 2024. Annual output ranged from 51 to 127 articles, with 973 publications from 2015 to 2024, representing a 45% increase compared to the preceding decade (672 publications from 2000 to 2014), indicating modest growth in research activity.

#### 3.1.3 Bibliometric analysis of articles

Table 1 lists the top 10 most cited articles in the field over the past 24 years (citation range: 482–1898). The most cited work, “Nutritional risk screening (NRS 2002): a new method based on an analysis of controlled clinical trials,” published in *Clinical Nutrition* by Kondrup in 2003, was the most cited article (1898 citations). Notably, two of the top-cited articles appeared in the *New England Journal of Medicine*.

**Table 1:** Top cited list of the top 10 highly cited papers related to renal function or nutritional indicators in patients with renal insufficiency treated with amino acids from 2000 to 2024.

Ranking	Authors	Year	Source title	Cited by
1st	Kondrup, J et al	2003	*Clinical Nutrition*	1,898
2nd	Bauer J, et al	2013	*Journal of the American Medical Directors Association*	1,573
3rd	Fanali G, et al	2012	*Molecular Aspects Of Medicine*	1,385
4th	Vanholder R, et al	2003	*Kidney International*	1,322
5th	Muscaritoli M, et al	2010	*Clinical Nutrition*	1,198
6th	Tepel M, et al	2000	*New England Journal of Medicine*	1,090
7th	Mangano DT, et al	2006	*New England Journal of Medicine*	803
8th	Carrero JJ, et al	2013	*Journal of Renal Nutrition*	602
9th	Gheorghiade M, et al	2004	*Journal of the American Medical Association*	495
10th	Ikizler TA, et al	2013	*Kidney International*	482

#### 3.1.4 Bibliometric analysis of authors

The 1948 publications involved 11,354 authors, with the top 66 contributors (≥5 publications each) representing 0.58% of all authors. The top 10 authors with the most publications and the top 10 most cited authors in the field of AA on renal function and nutrition in patients with RI from 2000 to 2024 are shown in Table 2. Kamyar Kalantar-Zadeh led in both productivity (23 articles) and citations (2693).

**Table 2:** Top 10 prolific authors and top 10 most cited authors on renal function or nutritional indicators in patients with renal insufficiency treated with amino acids from 2000 to 2024.

Rank	Author	H-index	G-index	M-index	TC	NP	PY start
1st	Kalantar-Zadeh K.	19	23	1.000	2,693	23	2007
2nd	Kopple Joel D.	14	15	0.700	1,031	15	2006
3rd	Talat Alp I.	13	13	0.650	1,701	13	2006
4th	Stenvinkel Petter	11	12	0.579	2,026	12	2007
5th	Bakker Stephan J. L.	6	12	0.316	235	12	2007
6th	Garibotto Giacomo	10	11	0.526	683	11	2007
7th	Cupisti Adamasco	10	10	0.588	456	10	2009
8th	Garneata Liliana	6	10	0.429	386	10	2012
9th	Fiaccadori Enrico	9	9	0.500	410	9	2008
10th	Kovesdy Csaba P.	9	9	0.500	969	9	2008
1st	Kalantar-Zadeh K.	19	23	1.000	2,693	23	2007
2nd	Zidek Walter	2	2	0.077	2,412	2	2000
3rd	Teta Daniel	6	6	0.333	2,227	6	2008
4th	Stenvinkel Petter	11	12	0.579	2,026	12	2007
5th	Kondrup Jens C.	1	1	0.043	1,898	1	2003
6th	Talat Alp I.	13	13	0.650	1,701	13	2006
7th	Passlick Deetjen J.	2	2	0.087	1,628	2	2003
8th	Wanner C.	4	4	0.154	1,614	4	2000
9th	Brunet Philippe	2	2	0.087	1,594	2	2003
10th	Boirie Yves	2	2	0.154	1,576	2	2013

TC, total cited; NP, number of publications; PY start, publication year start.

#### 3.1.5 Bibliometric analysis of journals

**Figure 2:**
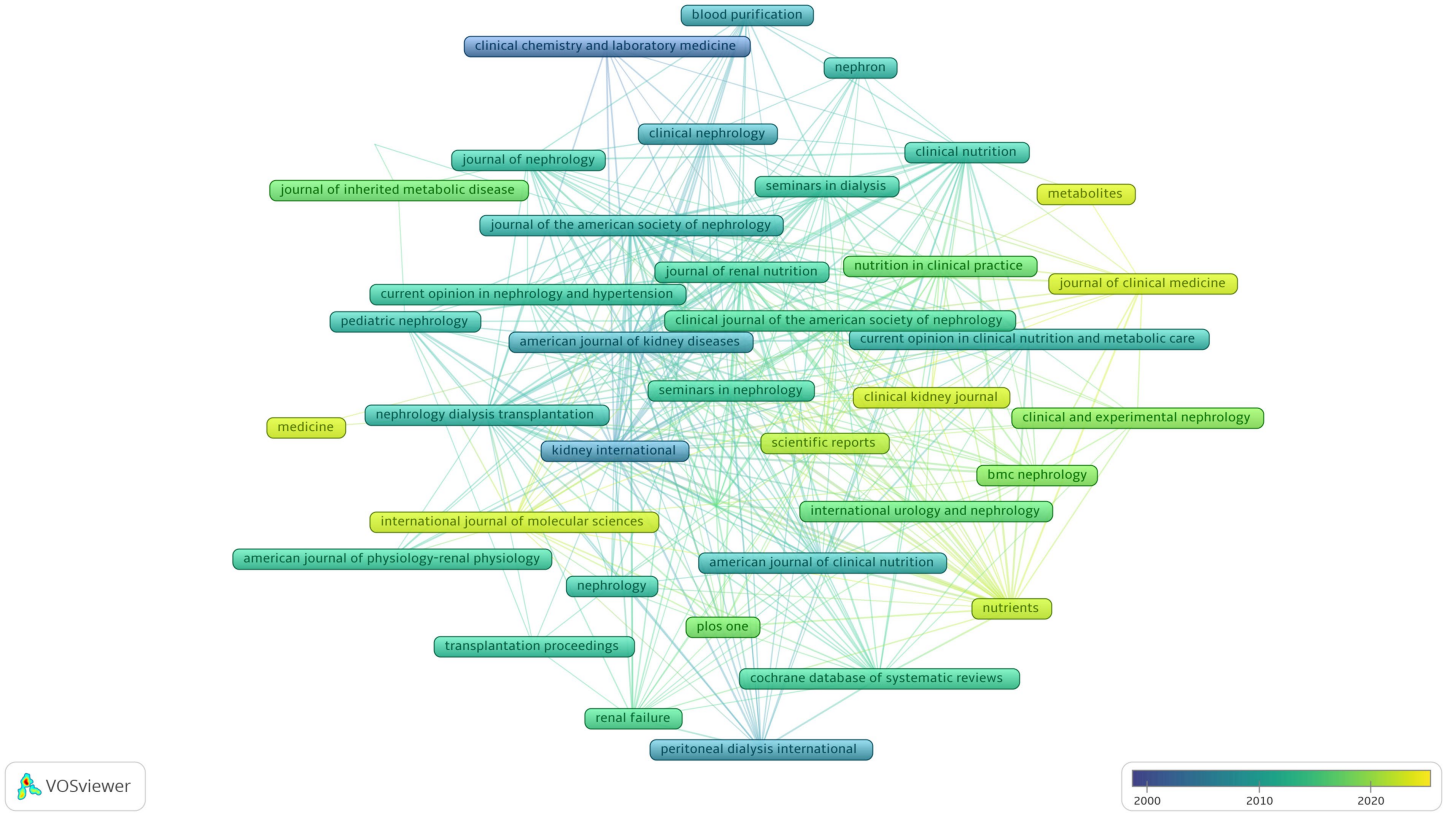
Evolution of journals over time plotted with VOSviewer. In the visualization map, the color of the label reflects the sequence of the appearance of a journals. The yellower the color of a journals was, the later it appeared, and the bluer the color of a journals was, the earlier the journals appeared.

Table 3 lists the top 10 journals with the most publications related to the field of renal function and nutrition in patients with AA and RI. *Kidney International* ranked first (65 articles, 3.34%), followed by the *Journal of Renal Nutrition* (53 articles, 2.72%). Figure 2 shows a trend graph of the evolution of journals in this field over time.

**Table 3:** List of the top 10 journals publishing research on renal function or nutritional indicators in patients with renal insufficiency treated with amino acids from 2000 to 2024.

Ranking	Journal	Frequency	%	IFa
1st	*Kidney International*	65	3.34	14.8
2nd	*Journal of Renal Nutrition*	53	2.72	3.4
3rd	*American Journal of Kidney Diseases*	51	2.62	9.4
4th	*Nephrology Dialysis Transplantation*	47	2.41	4.8
5th	*Nutrients*	41	2.10	4.8
6th	*Renal Failure*	34	1.75	3.1
7th	*BMC Nephrology*	31	1.59	2.2
8th	*PLoS ONE*	31	1.59	2.9
9th	*Clinical Nephrology*	28	1.44	1.1
10th	*Transplantation Proceedings*	28	1.44	0.8

SCR, standard competition ranking; IF, impact factor. An impact factors based on the 2023 journal citation reports 2023 from Clarivate analytics.

#### 3.1.6 Bibliometric analysis of countries

Over the past 24 years, at least 86 different countries have been involved in the publication of research in the field of renal function and nutrition in patients with AA and RI. The top 10 countries have authored a total of 1,387 articles, accounting for 71.2% of all studies in the relevant field. Table 4 shows the ranking of production and intercountry collaboration by author’s country, with the United States having the highest number of global publications in this field. Supplementary Figure S2 shows a map of national or regional collaborations in the field of renal function and nutrition in AA and RI between the major participating countries from 2000 to 2024. Table 5 shows the country citation analysis.

**Table 4:** Top 10 countries ranked by collaborations based on corresponding authors.

Rank	Country/Region	Articles	SCP	MCP	MCP%
1st	USA	385	297	88	22.9
2nd	China	260	228	32	12.3
3rd	Italy	171	142	29	17.0
4th	Japan	143	136	7	4.9
5th	Germany	125	77	48	38.4
6th	France	78	56	22	28.2
7th	UK	77	50	27	35.1
8th	Netherlands	55	42	13	23.6
9th	Sweden	47	34	13	27.7
10th	Turkey	46	42	4	8.7

SCP, single country publication; MCP, multi-country publication. Country represent the affiliation of the corresponding author. Articles denote the number of publications per country based on the corresponding author’s affiliation.

**Table 5:** Top 10 most cited countries on renal function or nutritional indicators in patients with renal insufficiency treated with amino acids from 2000 to 2024.

Rank	Country/region	Total cited	Average article citations
1st	USA	20,201	52.5
2nd	Italy	8,452	49.4
3rd	Germany	7,330	58.6
4th	China	4,092	15.7
5th	France	3,192	40.9
6th	Japan	3,084	21.6
7th	UK	2,943	38.2
8th	Belgium	2,924	132.9
9th	Denmark	2,565	106.9
10th	Sweden	2,194	46.7

#### 3.1.7 Bibliometric analysis of institutions

The University of California System had the highest number of publication-related studies among institutions worldwide, with 139 articles, or 7.14% of all articles. Harvard University was the second most prolific research institution with 95 articles (4.88%), followed by the University of California Los Angeles with 89 (4.57%) articles (Table 6).

**Table 6:** Top 10 prolific institutions in publishing papers on renal function or nutritional indicators in patients with renal insufficiency treated with amino acids from 2000 to 2024.

Rank	Institution	Documents	Country	% *N* = 1948
1st	University of California System	139	USA	7.14
2nd	Harvard University	95	USA	4.88
3rd	University of California Los Angeles	89	USA	4.57
4th	Karolinska Institutet	87	Sweden	4.47
5th	Institut national Sante Recherche Medicale (INSERM)	80	France	4.11
6th	University of London	77	UK	3.95
7th	Universite Paris Cite	72	France	3.70
8th	Egyptian Knowledge Bank (EKB)	67	Egypt	3.44
9th	Assistance Publique Hopitaux Paris (APHP)	65	France	3.34
10th	Vanderbilt University	63	USA	3.23

#### 3.1.8 Bibliometric analysis of keywords

**Figure 3:**
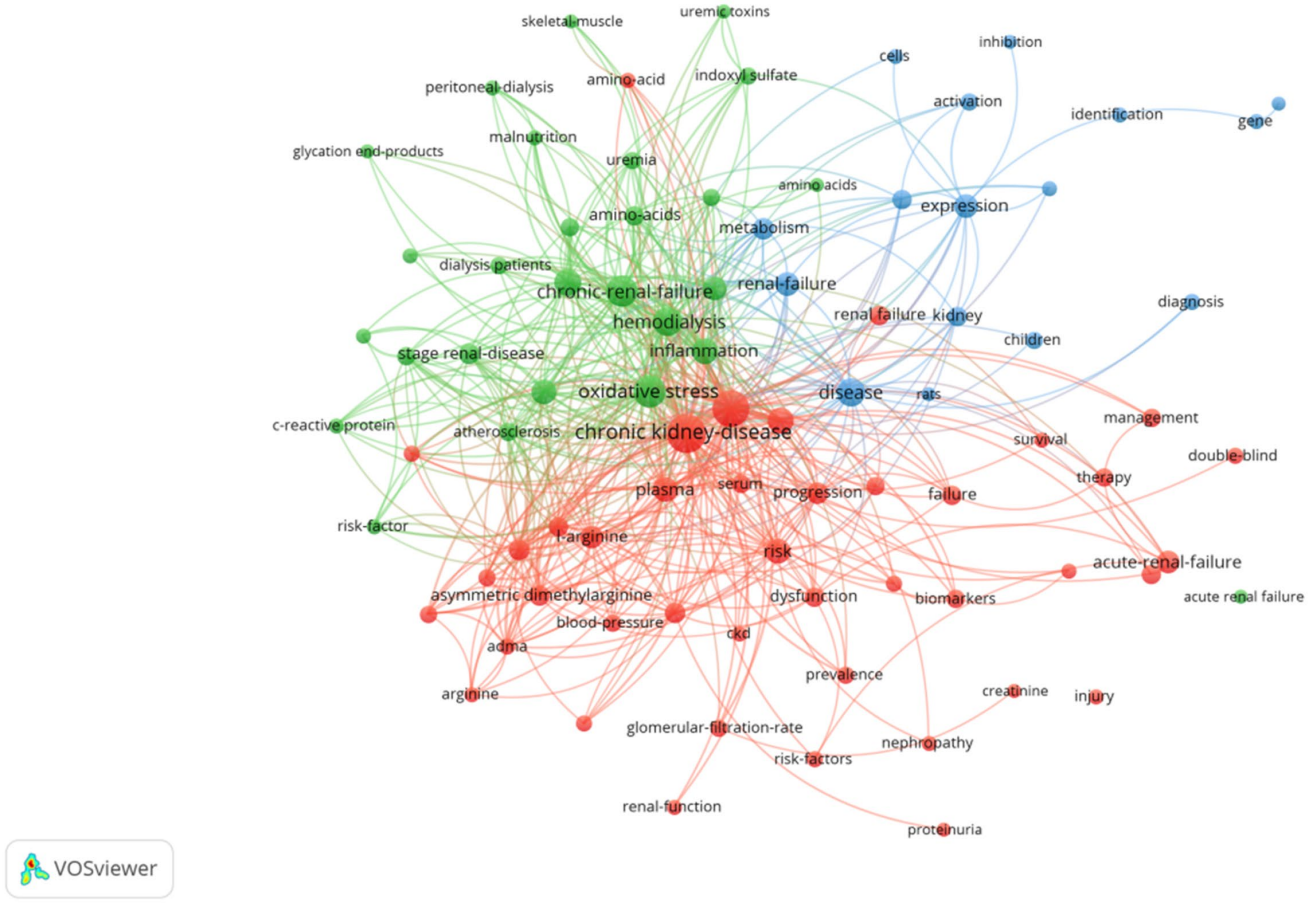
Network visualization map of terms in title/abstract fields of publications related to renal insufficiency and amino acids from 2000 to 2024. This visualized map of terms was developed when the minimum-term occurrences were placed at least 50 times. There are 85 terms that reach this threshold out of 8,497 in this field, which were divided into three clusters and colored differently. The size of the node indicates how many publications use that term.

**Figure 4:**
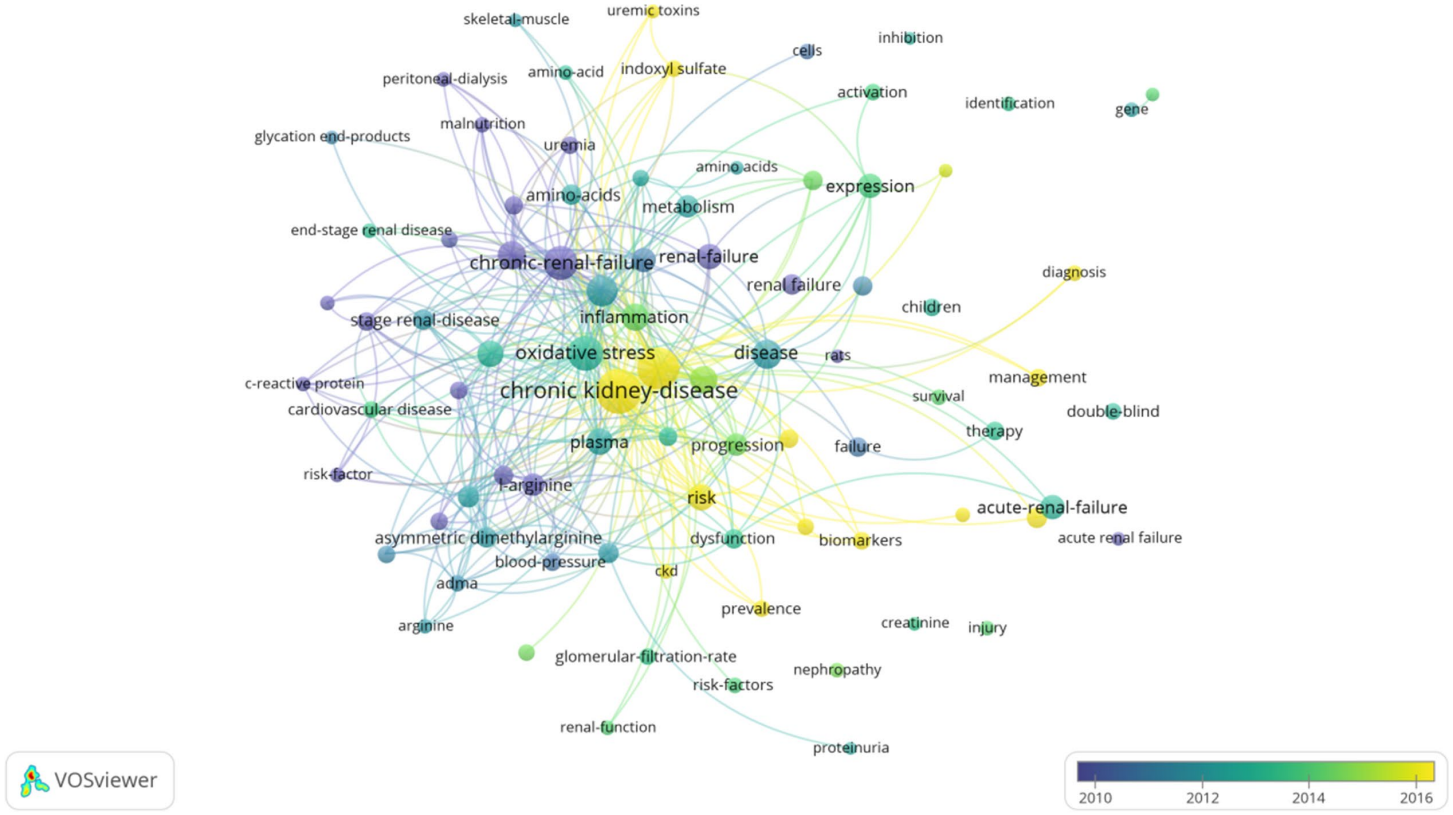
Network visualization map of terms in the title/abstract and their distribution according to the mean frequency of appearance. The blue terms emerged first, followed by the yellow and green terms that appeared later.

Terms with a minimum number of occurrences greater than 50 in all included publications were analyzed using the VOS viewer. Out of 8,497 terms, 85 terms reached this threshold, clustered into three thematic groups (Figure 3): Cluster 1 (Green): oxidative stress, chronic renal failure, hemodialysis, inflammation; Cluster 2 (Red): chronic kidney disease, risk, plasma, disease progression, L-arginine; Cluster 3 (Blue): renal failure, metabolism, kidney disease, biomarkers. Temporal keyword analysis revealed emerging foci on CKD, risk, biomarkers, and prevalence, suggesting future research directions (Figure 4).

### 3.2 Meta-analysis results

#### 3.2.1 Search results and study characteristics

Following systematic screening, 1,293 titles and abstracts of the literature were selected for further evaluation with 68 studies selected for full-text review. Ultimately, nine RCTs met the eligibility criteria and were included in the meta-analysis (11, 21–23, 34–38). Among the included studies: 7 RCTs reported ALB levels; 5 RCTs reported BUN; 4 RCTs provided TP data; 4 RCTs reported TRF; 3 RCTs included BMI, RPF, GFR and UA measurements. Table 7 provides a comprehensive overview of the specific details and population characteristics of the included studies. A total of 407 patients were included in the meta-analysis, of which 245 (60.2%) were dialysis patients (80 hemodialysis (HD) and 165 peritoneal dialysis (PD)), 63 (15.5%) chronic renal failure (CRF) patients, 54 (13.3%) renal transplant patients, and 45 (11%) glomerular injury patients. The cohort comprised 206 participants in intervention groups and 201 in control groups.

**Table 7:** Baseline demographics and characteristics of included RCTs.

Author, year	Countries	Participants (intervention/control)	Patients type	Mean age	Sex (F/M)	AA formulation	Comparator	Intervention time	Duration of intervention	Outcomes
Murtas, 2024 (22)	Italy	29 (14/15)	Hemodialysis	73.2	15/14	AminoDyal	Placebo	Novel mixture three times a week containing 5.4 g of AAs	3 months	BUN; UA; ALB; TP; TRF; BMI
Murtas, 2022 (11)	Italy	22 (11/11)	Hemodialysis	67.7	13/9	Amino-Ther-PRO	Placebo	A weekly total of 31.5 g AAs was given	6 months	BUN; UA; ALB; TP; TRF; BMI
Bolasco, 2011 (23)	Italy	29 (15/14)	Hemodialysis	73.9	19/10	Aminotrophic	Standard care	oral amino acid supplementation (4 g thrice a day)	3 months	BUN; ALB; TP; BMI
Hladunewich, 2006 (21)	Canada	45 (22/23)	Glomerular injury	28.5	45/0	L-arginine	Placebo	(3.5 g every 6 h), or intravenously (10 g every 8 h)	10 days	RPF; GFR; ALB
Miller, 2003 (35)	Israel	42 (21/21)	Chronic renal failure	70.5	11/31	L-arginine	Placebo	Prior to the angiographic examination (20 g)	Over20–30 min	BUN
Li, 2003 (37)	China	60 (30/30)	Peritoneal dialysis	46.0	28/32	Nutrineal	Glucose	received one 2-L bag of Nutrineal (1.1% Total Amino Acids) every morning	3 years	ALB; TRF
Schramm, 2002 (34)	Germany	54 (28/26)	Kidney transplantation	47.4	19/35	L-arginine	Placebo (saline)	0.75 g/kg body weight/day	3 days	GFR; RPF
Nicola, 1999 (36)	Italy	21 (11/10)	Chronic renal failure	47.9	6/15	L-arginine	Placebo	0.2 g/kg body weight/day	6 months	BUN; RPF; GFR; ALB
Jones, 1998 (38)	United States	105 (54/51)	Peritoneal dialysis	53.5	56/49	Nutrineal	Glucose	Received one 2-L bag of Nutrineal (1.1% Total Amino Acids) every morning	3 months	UA; ALB; TP; TRF

AA, amino acids; BUN, blood urea nitrogen; RPF, renal plasma flow; GFR, glomerular filtration rate; UA, uric acid; ALB, albumin; TP, total protein; TRF, transferrin; BMI, body mass index.

#### 3.2.2 Quality of enrolled trials

All 9 studies described random allocation methods, with 7 explicitly implementing allocation concealment and blinding protocols. Completeness of outcome data was reported in all studies, and none exhibited selective reporting. Based on the Cochrane RoB 2 tool, 7 studies were classified as low-risk and 2 as high-risk for bias (Supplementary Figure S3).

#### 3.2.3 Publication bias

Publication bias was assessed via funnel plots and Egger’s linear regression test. The funnel plot indicated no significant bias for ALB and BUN outcomes (Supplementary Figures S4, S5). The results of the remaining studies were assessed using the Egger test, and no significant bias was found either.

#### 3.2.4 BUN

**Figure 5:**
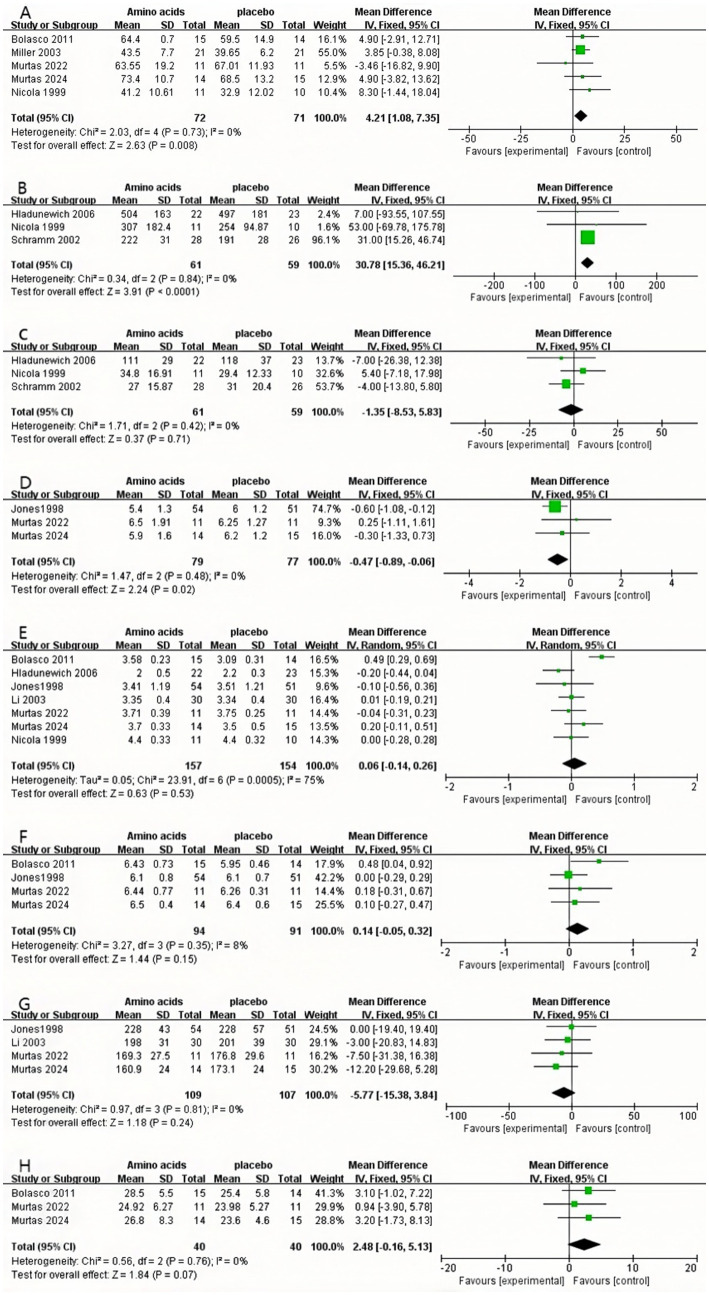
Forest plots of included RCTs. **(A)** Pooled analysis of RCTs evaluating the effect of amino acid therapy on BUN.·**(B)** Pooled analysis of RCTs evaluating the effect of amino acid therapy on RPF. **(C)** Pooled analysis of RCTs evaluating the effect of amino acid therapy on GFR. **(D)** Pooled analysis of RCTs evaluating the effect of amino acid therapy on UA. **(E)** Pooled analysis of RCTs evaluating the effect of amino acid therapy on ALB.·**(F)** Pooled analysis of RCTs evaluating the effect of amino acid therapy on TP. **(G)** Pooled analysis of RCTs evaluating the effect of amino acid therapy on TRF. **(H)** Pooled analysis of RCTs evaluating the effect of amino acid therapy on BMI.

The pooled estimates of the included trials demonstrated a statistically significant difference in BUN between the AA group and the control group (MD: 4.21, 95% CI: 1.08 to 7.35, *p* = 0.008, I^2^ = 0%, *N* = 5, fixed-effects model) (Figure 5A).

#### 3.2.5 RPF

The findings of the pooled estimates from the included trials demonstrated a statistically significant difference in RPF in the AA group compared with the control group (MD: 30.78, 95% CI: 15.36 to 46.21, *p* < 0.0001, I^2^ = 0%, *N* = 3, fixed-effects model) (Figure 5B).

#### 3.2.6 GFR

The findings of the pooled estimates from the included trials did not demonstrate a statistically significant difference in GFR in the AA group compared with the control group (MD: −1.35, 95% CI: −8.53 to 5.83, *p* = 0.71, I^2^ = 0%, *N* = 3, fixed-effects model) (Figure 5C).

#### 3.2.7 UA

The pooled estimates from the included trials demonstrated a statistically significant difference in UA in the AA group compared with the control group (MD: −0.47, 95% CI: −0.89 to −0.06, *p* = 0.02, I^2^ = 0%, *N* = 3, fixed-effects model) (Figure 5D).

#### 3.2.8 ALB

The results of the meta-analysis of the included trials revealed no statistically significant difference in ALB in the AA group in comparison with the control group (MD: 0.06, 95% CI: −0.14 to 0.26, *p* = 0.53, I^2^ = 75%, *N* = 7, random effects model) (Figure 5E).

#### 3.2.9 TP

The findings of the pooled estimates from the included trials indicated that there was no statistically significant difference in TP in the AA group compared with the control group (MD: 0.14, 95% CI: −0.05 to 0.32, *p* = 0.15, I^2^ = 8%, *N* = 4, fixed-effects model) (Figure 5F).

#### 3.2.10 TRF

The pooled estimates of the included trials demonstrated that there was no statistically significant difference in TRF in the AA group compared to the control group (MD: −5.77, 95% CI: −15.38 to 3.84, *p* = 0.24, I^2^ = 0%, *N* = 4, fixed-effects model) (Figure 5G).

#### 3.2.11 BMI

The pooled estimates from the included trials demonstrated that there was no statistically significant difference in BMI in the AA group compared with the control group (MD: 2.48, 95% CI: −0.16 to 5.13, *p* = 0.07, I^2^ = 0%, *N* = 3, fixed-effects model) (Figure 5H).

#### 3.2.12 Subgroup analysis

Given the substantial heterogeneity observed in ALB outcomes, subgroup analyses were conducted to evaluate potential confounding factors, including AA formulation type, RI subtype, and intervention duration (Supplementary Figure S6). These analyses revealed no statistically significant effect of AA formulation, RI classification, or treatment duration on ALB levels (*p* > 0.05).

#### 3.2.13 Sensitivity analysis

Sensitivity analyses of single studies were performed by exclusion, combining the remaining studies after excluding one study at a time to assess the impact of single studies on the combined results. The results showed that after the sequential exclusion of individual studies, there was no significant change in the confidence level among the remaining studies, indicating that the results of the meta-analysis were reliable (Supplementary material 3).

## 4 Discussion

Researchers with high citation rates or prolific publication outputs exert significant influence on the trajectory of scientific inquiry within a field, making their identification critical to understanding its leadership (39). This analysis identifies Kalantar-Zadeh Kamyar and Zidek Walter as pivotal contributors to advancing knowledge on AA interventions in RI. Understanding national and institutional contributions to research in this field will help researchers to seek collaborations between universities or international institutions to advance the field (40). For instance, the University of California System emerged as the most productive institution in this domain, underscoring its leadership in AA-related renal research.

Keyword and term analyses derived from article titles and abstracts reveal thematic priorities (41). Recurrent terms such as chronic kidney disease risk progression oxidative stress chronic renal failure inflammation renal failure expression and metabolism highlight a sustained focus on elucidating mechanisms underlying renal injury and failure progression. Density visualization emphasized chronic kidney disease and oxidative stress as dominant themes, while temporal trends identified biomarkers as an emerging keyword, reflecting intensified efforts to discover novel CKD biomarkers in recent years.

In terms of the development of research, the first study evaluating AA in RI was published in 1973, which showed that intravenous AA produced favorable effects in patients with acute renal insufficiency (42). Subsequent milestones include an RCT in 1982 comparing standard AA with essential amino acids (EAA) in renal impairment (43, 44), which solidified EAA supplementation as a sustained research priority in RI through 2000. Post-2000, investigations increasingly targeted CKD management, with recent emphasis on AA formulations to improve renal function and nutritional status in CKD and dialysis populations (11, 18). Notably, the role of AA in preventing cardiac surgery-associated acute kidney injury has emerged as a prominent focus, supported by high-quality clinical trials (10, 12, 45, 46).

Renal function indices were altered to varying degrees in patients with different types of RI compared to normal subjects. In normal physiology, intravenous AA administration elevates GFR via activation of renal functional reserve. However, this compensatory mechanism diminishes as CKD progresses to end-stage renal disease (47). Stevens et al. identified a sustained decline in GFR as a predictor of adverse outcomes in CKD (48). Seki et al. linked elevated BUN to accelerated renal deterioration in advanced CKD (stages 3–5) (49). Jiang et al. postulated that AA supplementation enhances RPF, thereby exerting renoprotective effects (50). Hyperuricemia and urinary stone formation, driven by elevated UA, are recognized risk factors for CKD progression (51, 52). Dysregulated AA metabolism contributes to hyperuricemia in CKD patients; AA supplementation may correct this imbalance and reduce UA levels, even in advanced disease (11, 53). This meta-analysis synthesizes evidence supporting AA’s potential renal benefits. For instance, there was a numerical increase in RPF in 3 of the 3 studies (11, 21, 36), and UA levels were reduced in 2 of the 3 studies (11, 38). Consistent with preliminary results and preclinical studies. However, BUN levels were elevated in 4 of 5 studies (22, 23, 35, 36). Singer et al. noted that short-term AA infusion (75 g/day) in acute kidney injury patients significantly raised BUN by days 2–4 (*p* < 0.04) (50), likely due to increased protein catabolism. In stable CKD, elevated BUN is primarily attributed to excessive protein intake (51), and low-protein diets effectively mitigate this effect (52). Therapeutic strategies may thus require tailored approaches: high-dose AA infusion (150 g/day) for short-term acute kidney injury management versus long-term protein restriction in CKD to counteract BUN elevation (54, 55).

Nutrition serves as a cornerstone in CKD management (56). The kidneys are central to maintaining nutritional homeostasis, regulating AA balance partially through synthesis and catabolism (57). In therapeutic contexts, AA is primarily utilized in drug therapy to support protein synthesis (58). For patients with kidney injury, 9AA is generally used, which contains eight EAA and histidine in its composition, and the intervention in this study contained 9AA in all AA mixtures except arginine (59). EAA supplementation in CKD patients aids in preserving protein homeostasis (19), while biomarkers such as ALB, TP, TRF, and BMI are widely employed to assess nutritional status in CKD and HD populations (60). Protein depletion and malnutrition are prevalent in HD-treated CKD patients due to AA losses during dialysis (17). Murtas et al. demonstrated that CKD disrupts AA kinetic metabolism, impairing protein anabolism, a phenomenon exacerbated by HD-induced AA depletion (11). Conversely, Erkan et al.’s retrospective study found no significant improvement in ALB levels with daily 1.1% AA solution use in PD patients over 12 months (61). This meta-analysis corroborates prior findings, revealing no statistically significant impact of AA therapy on ALB, TP, TRF, or BMI in RI patients (17, 62). The lack of significant nutritional parameter changes may be attributed to (1) Intervention heterogeneity: Included studies utilized diverse AA formulations (e.g., L-arginine, variable EAA compositions) and short intervention durations (7/9 studies ≤3 months), potentially blunting effects on slow-turnover biomarkers like ALB (half-life: 2–3 weeks) (63). (2) CKD-associated catabolic states: Uremic toxin accumulation, chronic inflammation, and dialysis-induced AA losses (up to 800 g/year in HD patients) counteract the anabolic effects of supplementation, with 60.2% of participants in the population included in this article receiving dialysis treatment (22, 64). These findings underscore a critical limitation: standalone nutritional parameters may not capture AA efficacy in complex RI populations, necessitating integrated assessments of inflammation, metabolic markers, and patient-centered outcomes. For CKD patients, especially those who need dialysis, supplemental AA therapy should be supplemented with attention to the nutritional status of the patients and appropriate nutritional supplements, while focusing on the changes in renal function indices for a protein-restricted diet or other effective measures. Future studies should further explore the effects of different AA types, dosages, and treatment durations on nutritional indicators to optimize the treatment regimen.

This study has several limitations. In the bibliometric analysis, reliance solely on the Web of Science database may introduce selection bias, as inclusion of additional repositories (e.g., Scopus, MEDLINE) could yield divergent results. Furthermore, the search strategy encompassed studies involving AA structural derivatives, potentially capturing marginally relevant literature. For the meta-analysis, heterogeneity in eligibility criteria, control interventions, AA treatment components (e.g., dosage, formulation), and patient subtypes (e.g., CKD stages, dialysis modalities) limits the generalizability of pooled results. Such variability underscores the need for standardized protocols in future trials to enhance comparability.

## 5 Conclusion

Bibliometric analysis suggests that the keywords oxidative stress, inflammation, risk, and progression aim to shed light on possible mechanisms of RI. Keyword trends focused on CKD and new biomarkers, findings that reiterated the importance of chronic kidney disease progression and mechanisms. The meta-analysis yielded a multifaceted therapeutic landscape for AA supplementation: while it exhibited promising effects by enhancing RPF and reducing UA levels, these advantages were counterbalanced by sustained elevation in BUN and lack of significant alterations in nutritional parameters. This suggests that the renal impacts of AA are context-dependent, influenced by factors such as patient population, intervention duration, and formulation composition, and thus warrant nuanced interpretation and judicious clinical consideration. Clinically, these findings underscore the need to balance observed improvements in GFR and UA against potential risks associated with BUN elevation when integrating AA supplementation into therapeutic regimens.

### 5.1 Future research priorities

Priority areas include the following to address existing evidence gaps: (1) Dose–response characterization: Conduct dose-ranging studies to define thresholds for optimizing renal protection while minimizing BUN elevation; (2) Mechanistic investigations: Elucidate interactions between AA formulations, uremic metabolism, and inflammatory pathways influencing renal and nutritional outcomes; (3) Stratified trial designs: Perform subgroup analyses by CKD stage (pre-dialysis vs. HD/PD) and AA subtype, complemented by adaptive dose-finding trials; and (4) Long-term outcome evaluation: Implement RCTs with ≥1-year follow-up to characterize AA therapy’s impact on end-stage renal disease progression and survival.
