# Correction: Impacts of amino acid supplementation on renal function and nutritional parameters in patients with renal insufficiency: bibliometric analysis and meta-analysis

**DOI:** 10.3389/fnut.2025.1760327

**Published:** 2026-01-12

**Authors:** Xiaoxia Liu, Qiufu Li, Liyuan Zhang, Ying He, Sitao Tan, Xiaoyu Chen, Kefeng Li

**Affiliations:** 1 Center for Artificial Intelligence Driven Drug Discovery, Faculty of Applied Sciences, Macao Polytechnic University, Macao, China; 2 Department of Pharmacy, The People's Hospital of Guangxi Zhuang Autonomous Region, Guangxi Academy of Medical Sciences, Nanning, China; 3 Phase 1 Clinical Trial Laboratory, The People's Hospital of Guangxi Zhuang Autonomous Region, Guangxi Academy of Medical Sciences, Nanning, China

**Keywords:** renal insufficiency, amino acids, meta-analysis, bibliometrics, renal function, nutritional indicators

In the published article, the incorrect grant number was provided for Macao Polytechnic University (RP/FCA-14/2023). The correct grant number is (RP/FCA-14/2023) with the submission approval ID: fca.aa3e.e0a8.4. The correct Funding Statement appears below.

The original version of this article has been updated.

