# Supplementary material for: Impacts of amino acid supplementation on renal function and nutritional parameters in patients with renal insufficiency: bibliometric analysis and meta-analysis
Source: Front Nutr. 2025 Jun 13;12:1594507. doi: 10.3389/fnut.2025.1594507 (PMC12202395; doi:10.3389/fnut.2025.1594507)
Supplement: Supplementary file 1 [file Table_1.docx]

*Supplementary Material 1*

**Web of science**

#1:TS="Kidney disorders" OR "Kidney injury" OR "Kidney diseases" OR "Kidney failure" OR "Kidney insufficiency" OR "Kidney impairment" OR "Kidney dysfunction" OR "Renal disorders" OR "Renal injury" OR "Renal diseases" OR "Renal failure" OR "Renal insufficiency" OR "Renal impairment" OR "Renal dysfunction"OR nephropathy OR "end-stage renal disease" OR "end-stage kidney disease" OR azotemia OR uremia OR "irreversible kidney failure" OR "dialysis" OR hemodialysis OR AKI OR AKD OR CKD OR CRI OR ARI OR ESKD OR ARF OR CRF

#2:TS="Amino acids" OR "Acids, Amino" OR "Amino Acid" OR "Acid, Amino" OR "Amino-Acid" OR glycine OR alanine OR valine OR leucine OR isoleucine OR methionine OR proline OR tryptophan OR serine OR tyrosine OR cysteine OR phenylalanine OR asparagine OR glutamine OR threonine OR "aspartic acid" OR "glutamic acid" OR lysine OR arginine OR histidine

#3:TS="patients" OR "patient" OR "patients" OR "Clients" OR "Client" OR "Human"

#4:TS="kidney function" OR "renal function" OR "function test, kidney" OR "function tests, kidney" OR "kidney function test" OR "test, kidney function" OR "tests, kidney function" OR nutrition OR nutritional

#5:NOT TS="Experimentation, Animal" OR "Animal Experimental Use" OR "Animal Experimental Uses" OR "Experimental Use, Animal" OR "Experimental Uses, Animal" OR "Animal Research" OR "Research, Animal" OR "Animal Experiments" OR "Animal Experiment" OR "Experiment, Animal" OR "Experiments, Animal" OR rat OR mouse OR mice OR fish OR dogs OR dog OR cat OR cats OR monkeys OR monkey

#6:#1 AND #2 AND #3 AND #4 AND #5

Timespan: 2000-01-01 to 2024-12-31 (Index Date)

Refined By:Languages: English

**Pumed:(1122)**

(("Kidney diseases"[MeSH Terms] OR "Kidney failure"[Title/Abstract] OR "Kidney disorders"[Title/Abstract] OR "Kidney injury"[Title/Abstract] OR "Kidney insufficiency"[Title/Abstract] OR "Kidney impairment"[Title/Abstract] OR "Kidney dysfunction"[Title/Abstract] OR "Renal disorders"[Title/Abstract] OR "Renal injury"[Title/Abstract] OR "Renal diseases"[Title/Abstract] OR "Renal failure"[Title/Abstract] OR "Renal insufficiency"[Title/Abstract] OR "Renal impairment"[Title/Abstract] OR "Renal dysfunction"[Title/Abstract] OR AKI[Title/Abstract] OR AKD[Title/Abstract] OR CKD[Title/Abstract] OR CRI[Title/Abstract] OR ARI[Title/Abstract] OR ESKD[Title/Abstract] OR ARF[Title/Abstract] OR CRF[Title/Abstract] OR nephropathy[Title/Abstract] OR "End-stage renal disease"[Title/Abstract] OR "End-stage kidney disease"[Title/Abstract] OR Azotemia[Title/Abstract] OR Uremia[Title/Abstract] OR "Irreversible kidney failure"[Title/Abstract] OR Dialysis[Title/Abstract] OR Hemodialysis[Title/Abstract]) AND ("Amino Acid"[Title/Abstract] OR "Amino Acids"[MeSH Terms] OR "Acids, Amino"[Title/Abstract] OR "Acid, Amino"[Title/Abstract] OR "Amino-Acid"[Title/Abstract] OR Glycine[Title/Abstract] OR alanine[Title/Abstract] OR valine[Title/Abstract] OR leucine[Title/Abstract] OR isoleucine[Title/Abstract] OR methionine[Title/Abstract] OR proline[Title/Abstract] OR tryptophan[Title/Abstract] OR serine[Title/Abstract] OR tyrosine[Title/Abstract] OR cysteine[Title/Abstract] OR phenylalanine[Title/Abstract] OR asparagine[Title/Abstract] OR glutamine[Title/Abstract] OR threonine[Title/Abstract] OR aspartic acid[Title/Abstract] OR glutamic acid[Title/Abstract] OR lysine[Title/Abstract] OR arginine[Title/Abstract] OR histidine[Title/Abstract])) AND ((randomized controlled trial[Filter]) AND (humans[Filter]) AND (english[Filter]))

**Embase:(489)**

#1:(((Acute:ab,ti OR Chronic:ab,ti) AND (kidney:ab,ti OR renal:ab,ti)) AND (disorders:ab,ti OR injury:ab,ti OR diseases:ab,ti OR failure:ab,ti OR insufficiency:ab,ti OR impairment:ab,ti OR dysfunction:ab,ti)) OR nephropathy:ab,ti OR 'end-stage renal disease':ab,ti OR 'end-stage kidney disease':ab,ti OR 'azotemia':ab,ti OR Uremia:ab,ti OR 'irreversible kidney failure':ab,ti OR Dialysis:ab,ti OR Hemodialysis:ab,ti OR AKI:ab,ti OR AKD:ab,ti OR CKD:ab,ti OR CRI:ab,ti OR ARI:ab,ti OR ESKD:ab,ti OR ARF:ab,ti OR CRF:ab,ti

#2:'amino acid':ab,ti OR 'Acids, Amino':ab,ti OR 'Amino Acid':ab,ti OR 'Acid, Amino':ab,ti OR 'Amino-Acid':ab,ti OR Glycine:ab,ti OR alanine:ab,ti OR valine:ab,ti OR leucine:ab,ti OR isoleucine:ab,ti OR methionine:ab,ti OR proline:ab,ti OR tryptophan:ab,ti OR serine:ab,ti OR tyrosine:ab,ti OR cysteine:ab,ti OR phenylalanine:ab,ti OR asparagine:ab,ti OR glutamine:ab,ti OR threonine:ab,ti OR 'aspartic acid':ab,ti OR 'glutamic acid':ab,ti OR lysine:ab,ti OR arginine:ab,ti OR histidine:ab,ti

#3:'randomized controlled trial'/de

#4: #1 AND #2 AND #3

**Cochrane Library:(538)**

#1:MeSH descriptor: [Renal Insufficiency] explode all trees

#2:(Kidney disorders):ti,ab,kw OR (Kidney injury):ti,ab,kw OR (Kidney diseases):ti,ab,kw OR (Kidney failure):ti,ab,kw OR (Kidney insufficiency):ti,ab,kw OR (Kidney impairment):ti,ab,kw OR (Kidney dysfunction):ti,ab,kw OR (Renal disorders):ti,ab,kw OR (Renal injury):ti,ab,kw OR (Renal diseases):ti,ab,kw OR (Renal failure):ti,ab,kw OR (Renal insufficiency):ti,ab,kw OR (Renal impairment):ti,ab,kw OR (Renal dysfunction):ti,ab,kw OR (nephropathy):ti,ab,kw OR (End-stage renal disease):ti,ab,kw OR (End-stage kidney disease):ti,ab,kw OR (Azotemia):ti,ab,kw OR (Uremia):ti,ab,kw OR (Irreversible kidney failure):ti,ab,kw OR (Dialysis):ti,ab,kw OR (Hemodialysis):ti,ab,kw

#3:#1 OR #2

#4:MeSH descriptor: [Amino Acids] explode all trees

#5:(Amino Acid):ti,ab,kw OR (Acids, Amino):ti,ab,kw OR (Acid, Amino):ti,ab,kw OR (Amino-Acid):ti,ab,kw OR (Glycine):ti,ab,kw OR (alanine):ti,ab,kw OR (valine):ti,ab,kw OR (leucine):ti,ab,kw OR (isoleucine):ti,ab,kw OR (methionine):ti,ab,kw OR (proline):ti,ab,kw OR (tryptophan):ti,ab,kw OR (serine):ti,ab,kw OR (tyrosine):ti,ab,kw OR (cysteine):ti,ab,kw OR (phenylalanine):ti,ab,kw OR (asparagine):ti,ab,kw OR (glutamine):ti,ab,kw OR (threonine):ti,ab,kw OR (aspartic acid):ti,ab,kw OR (glutamic acid):ti,ab,kw OR (lysine):ti,ab,kw OR (arginine):ti,ab,kw OR (histidine):ti,ab,kw

#6: #4 OR #5

#7:#3 AND #6

#8:Randomized Clinical Trials:ti,ab,kw OR RCT:ti,ab,kw

#9:#7 AND #8
