# Supplementary material for: Impacts of amino acid supplementation on renal function and nutritional parameters in patients with renal insufficiency: bibliometric analysis and meta-analysis
Source: Front Nutr. 2025 Jun 13;12:1594507. doi: 10.3389/fnut.2025.1594507 (PMC12202395; doi:10.3389/fnut.2025.1594507)
Supplement: Supplementary file 2 [file Table_2.docx]

*Supplementary Material 2*


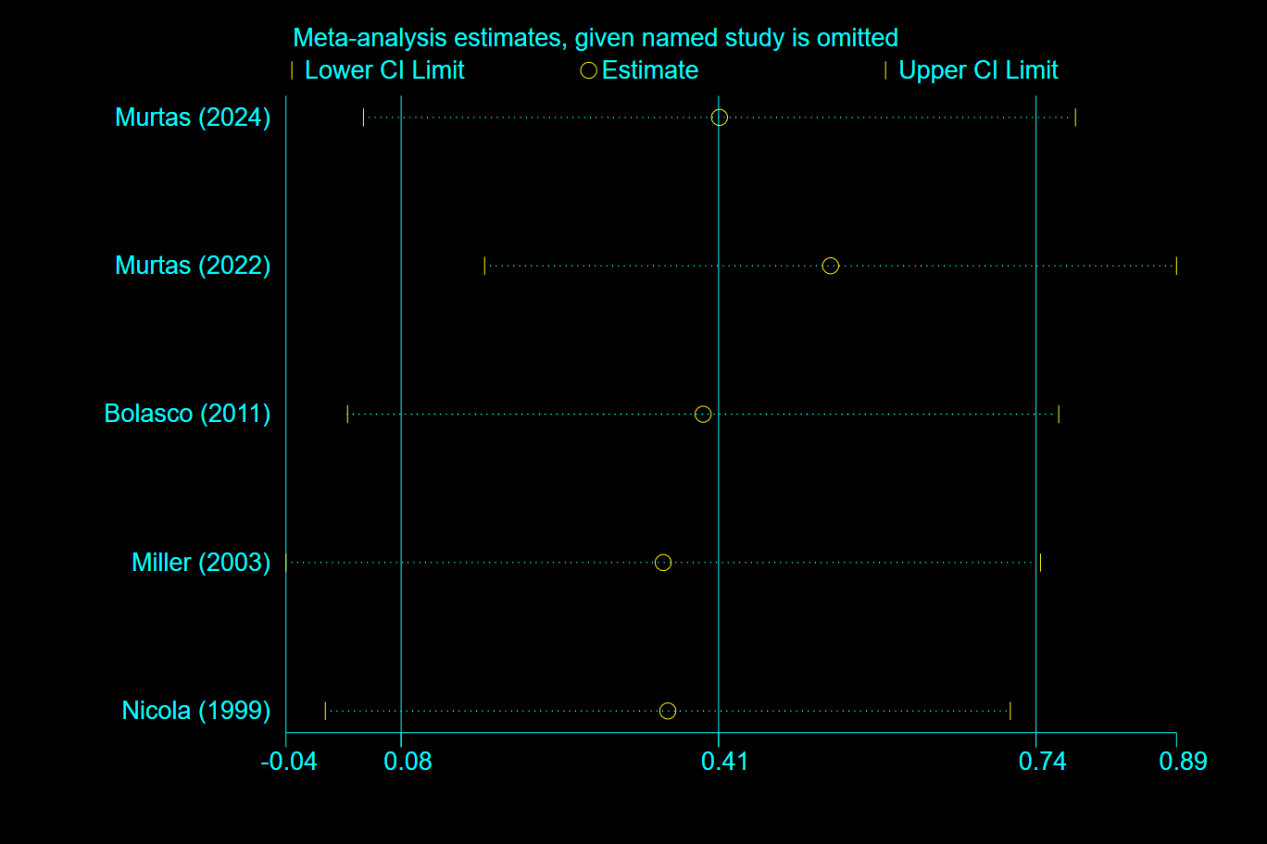


Sensitivity analyses of RCTs evaluating the effect of amino acid therapy on BUN in patients with RI.


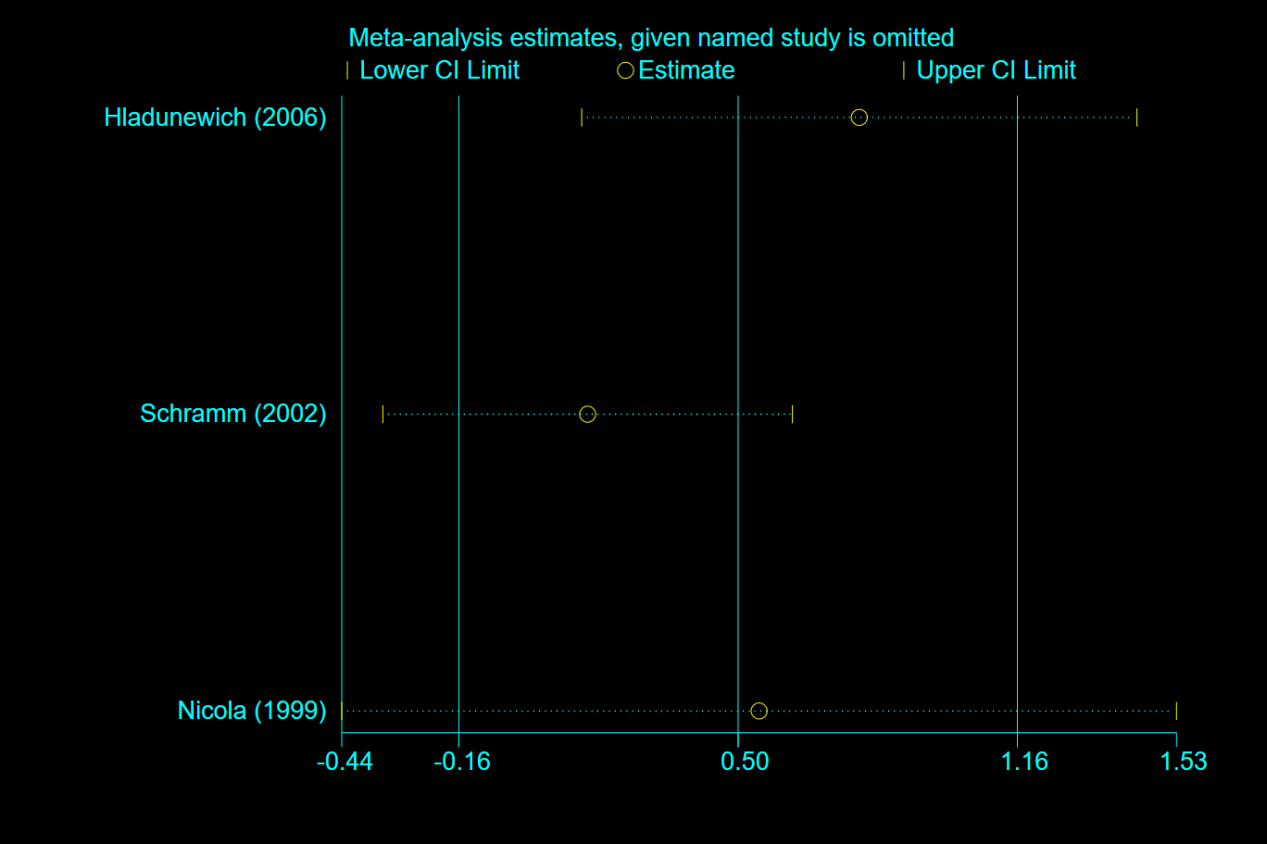


Sensitivity analyses of RCTs evaluating the effect of amino acid therapy on RPF in patients with RI.


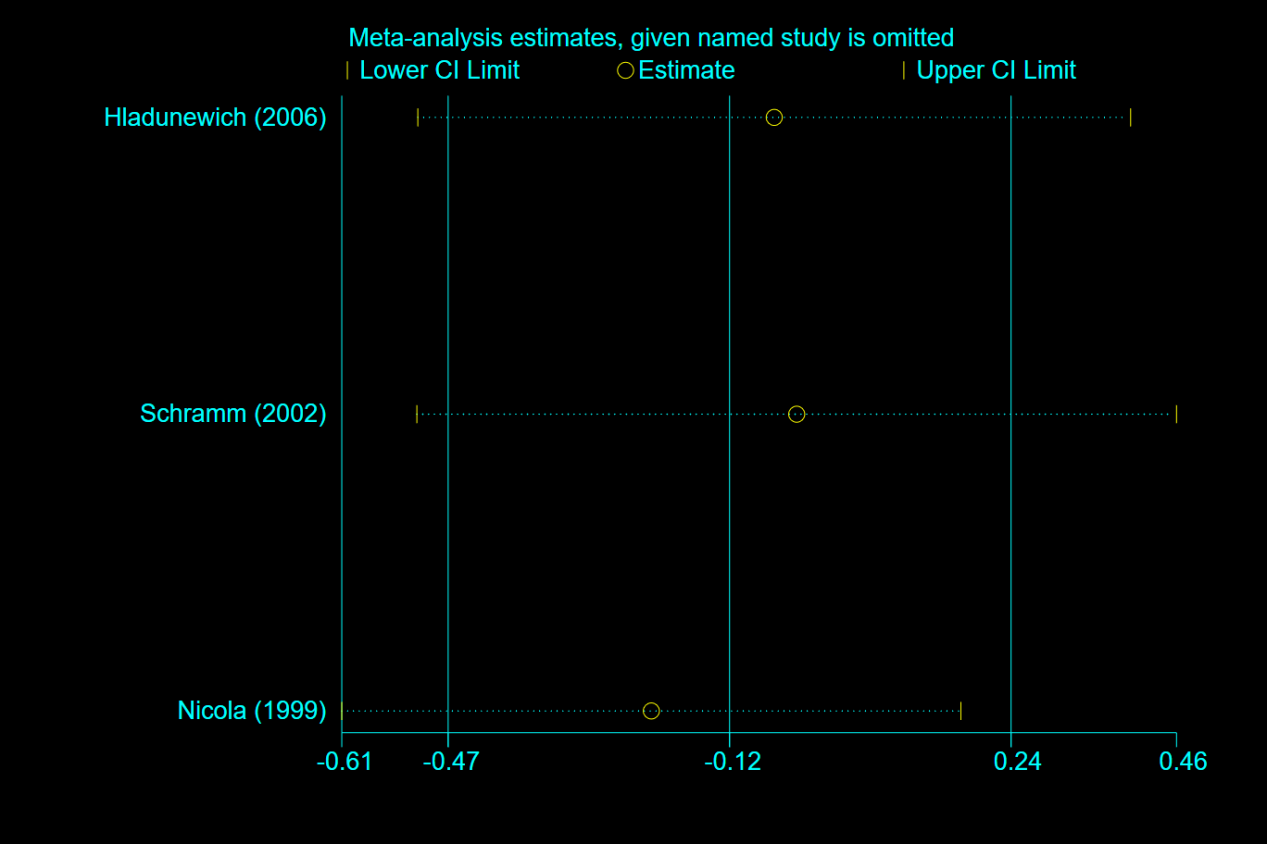


Sensitivity analyses of RCTs evaluating the effect of amino acid therapy on GFR in patients with RI.


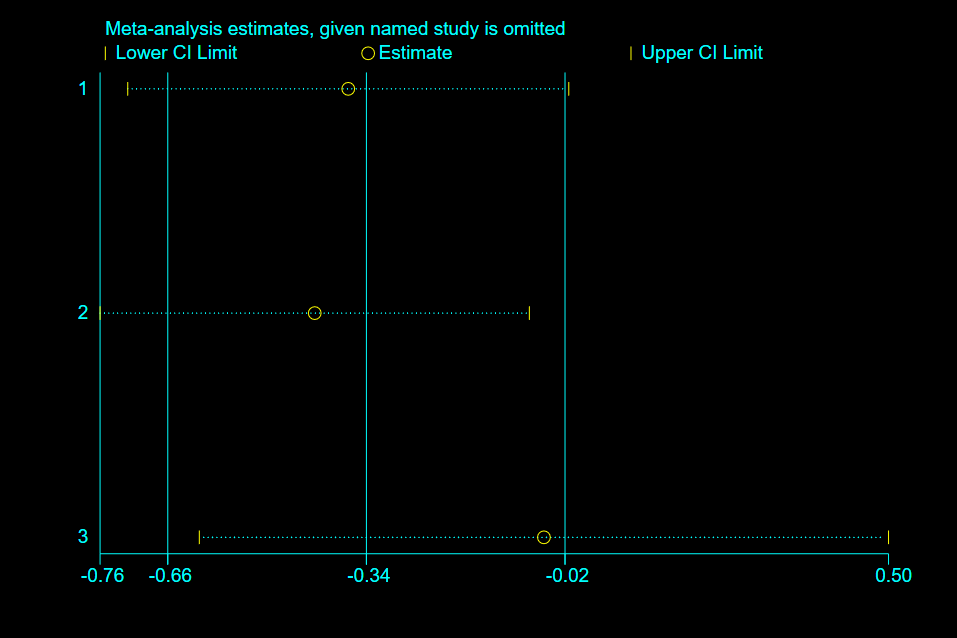


Sensitivity analyses of RCTs evaluating the effect of amino acid therapy on UA in patients with RI.


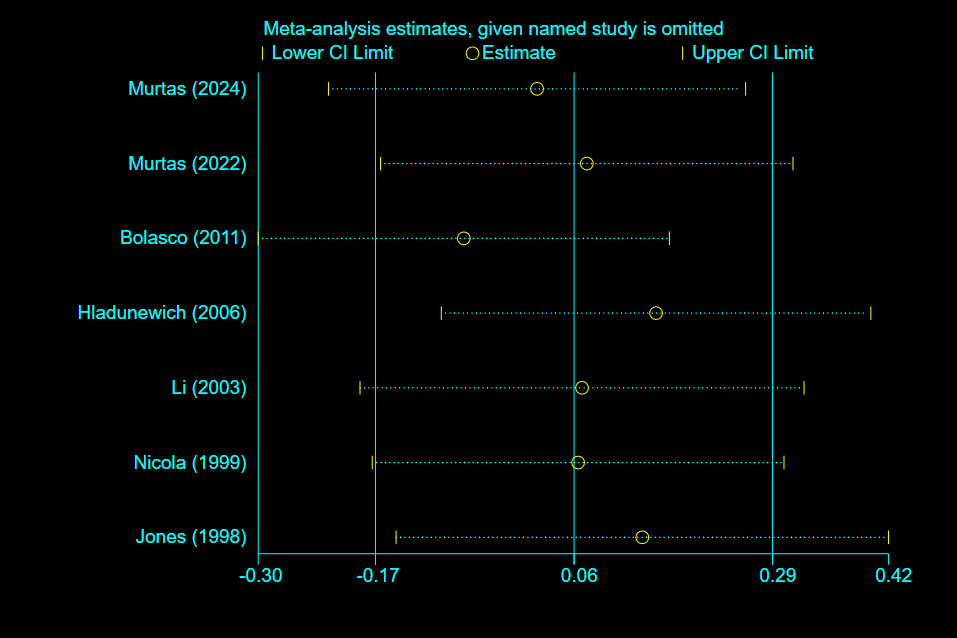


Sensitivity analyses of RCTs evaluating the effect of amino acid therapy on ALB in patients with RI.


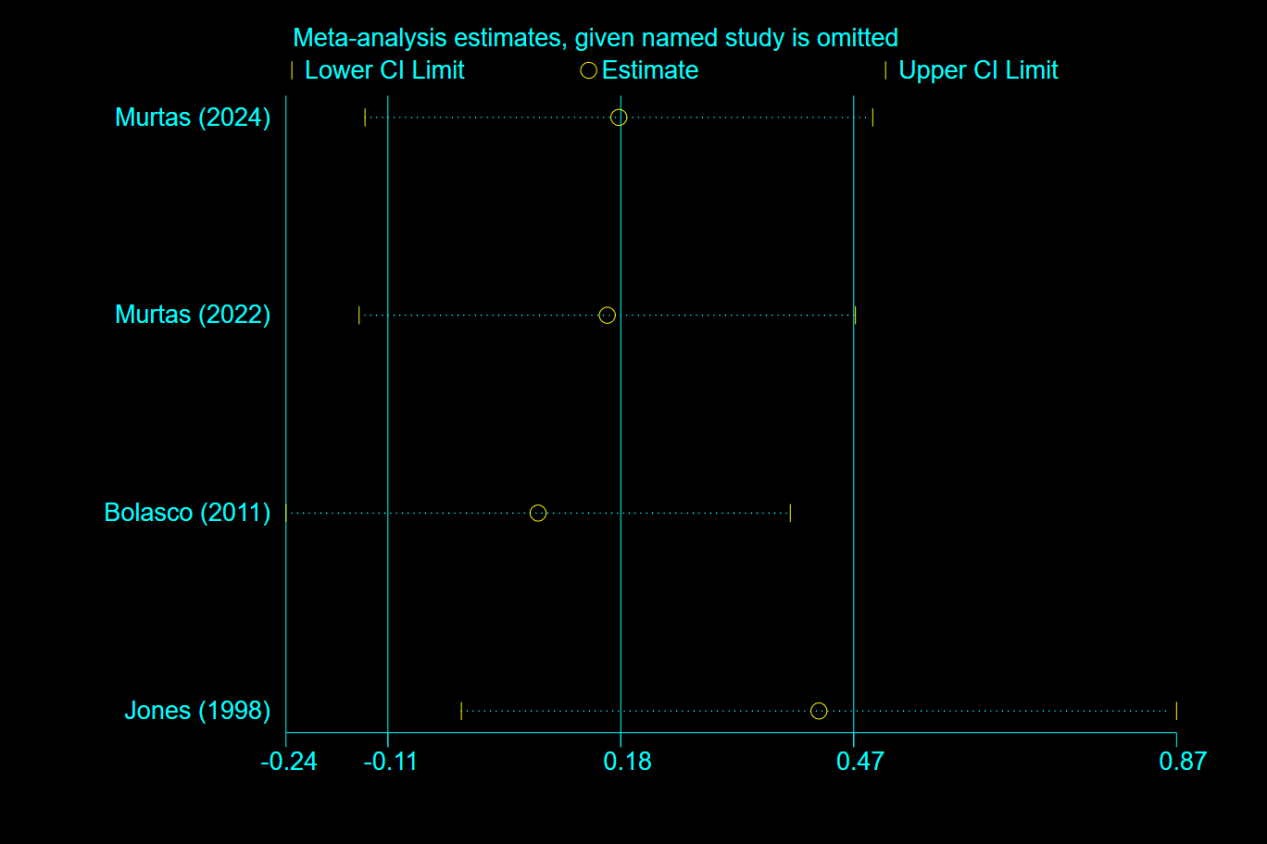


Sensitivity analyses of RCTs evaluating the effect of amino acid therapy on TP in patients with RI.


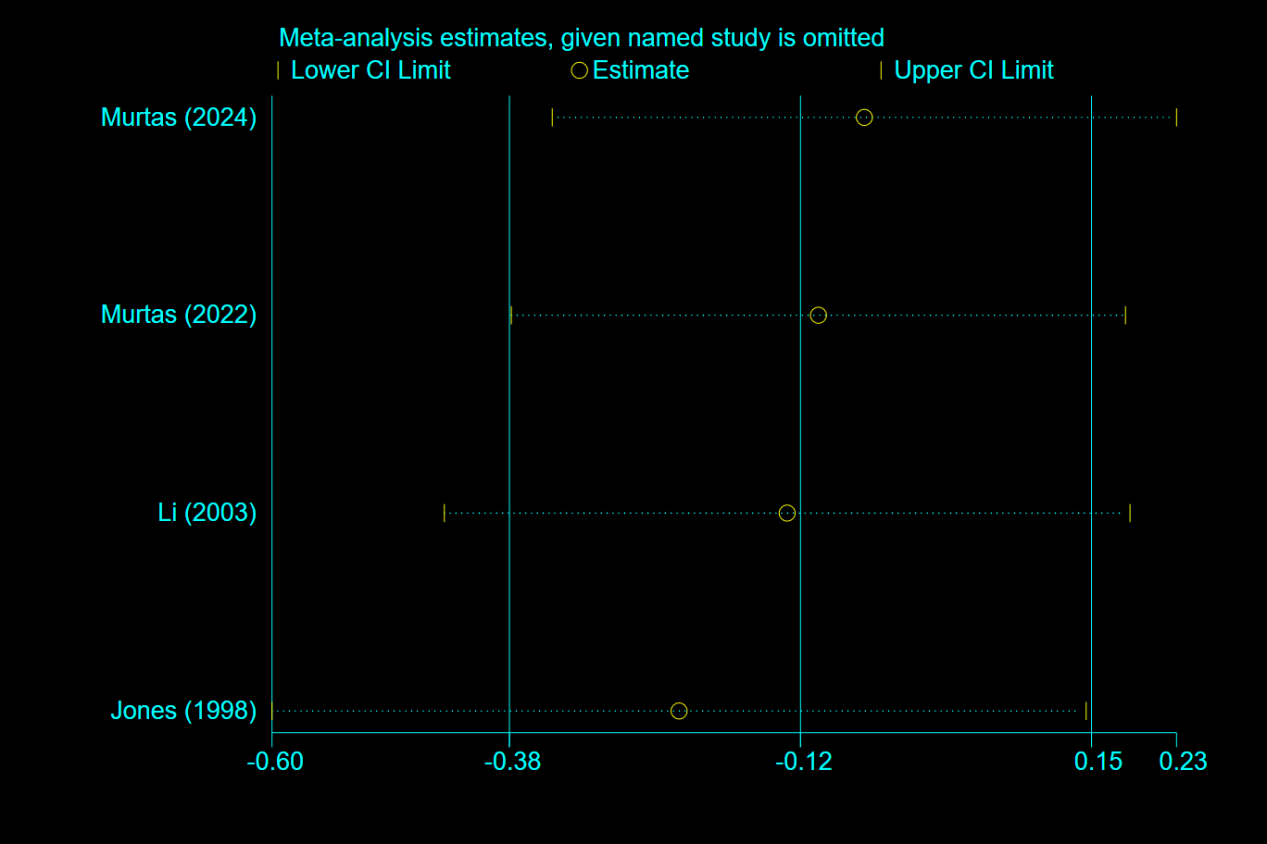


Sensitivity analyses of RCTs evaluating the effect of amino acid therapy on TRF in patients with RI.


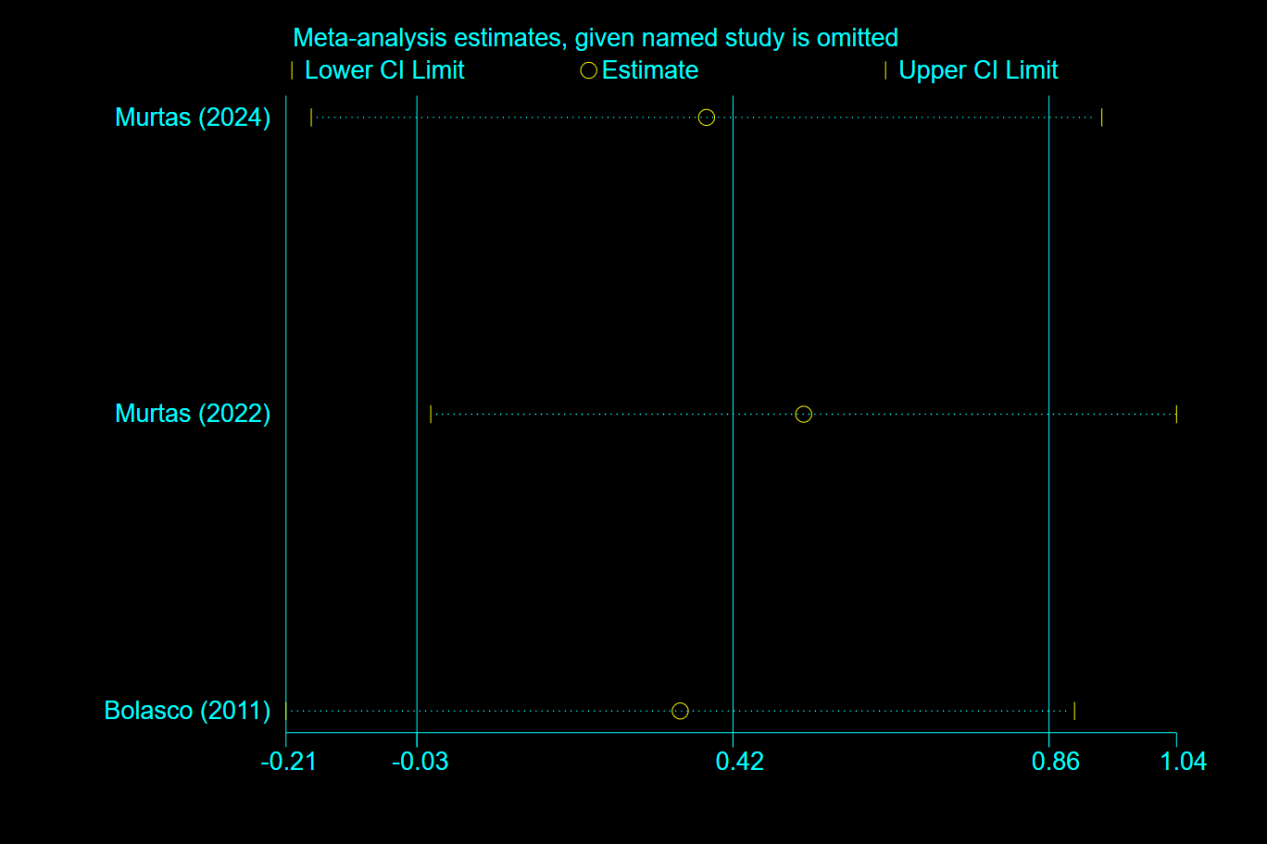


Sensitivity analyses of RCTs evaluating the effect of amino acid therapy on BMI in patients with RI.
