# Supplementary material for: Impacts of amino acid supplementation on renal function and nutritional parameters in patients with renal insufficiency: bibliometric analysis and meta-analysis
Source: Front Nutr. 2025 Jun 13;12:1594507. doi: 10.3389/fnut.2025.1594507 (PMC12202395; doi:10.3389/fnut.2025.1594507)
Supplement: Supplementary file 3 [file Image_1.pdf]

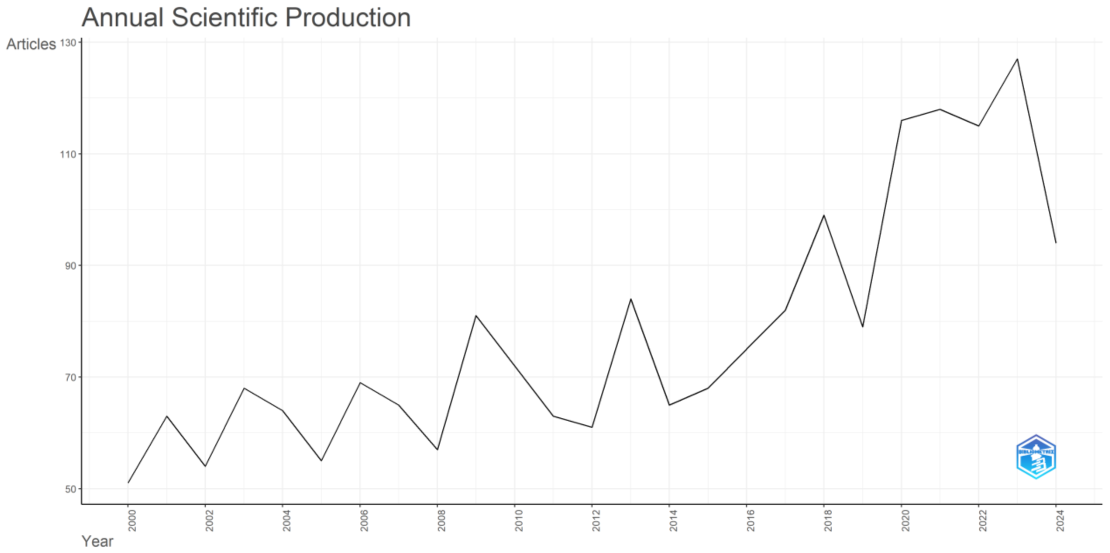

Fig.S1 Growth trend of publications related to the impact of amino acids on renal function or nutritional indicators in patients with renal insufficiency from 2000 to 2024.

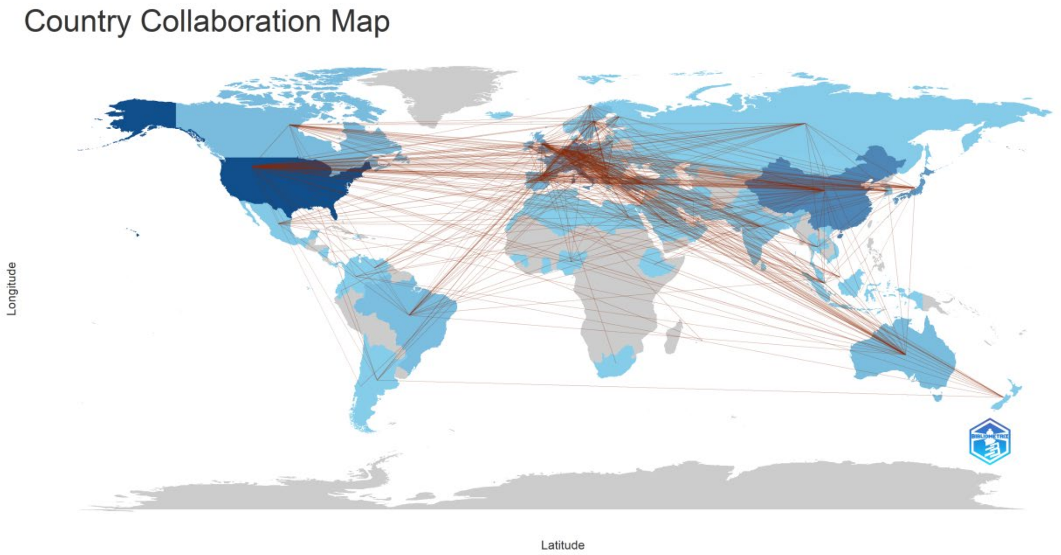

Fig.S2 Trends in the publications in renal insufficiency and amino acids research involved 86 countries/regions. The interconnection among nations signifies collaborative efforts.

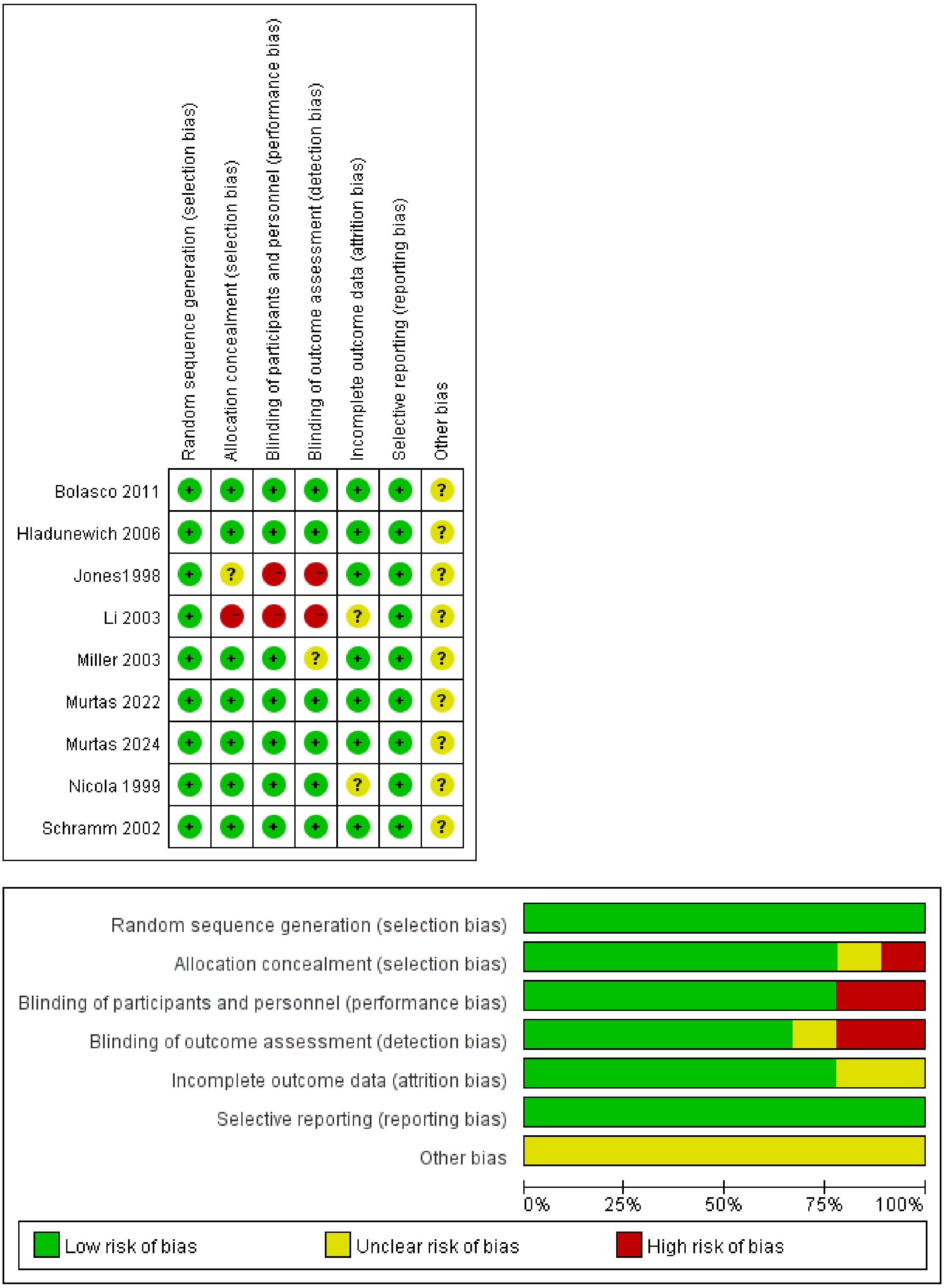

Fig.S3 Quality Assessment Charts.

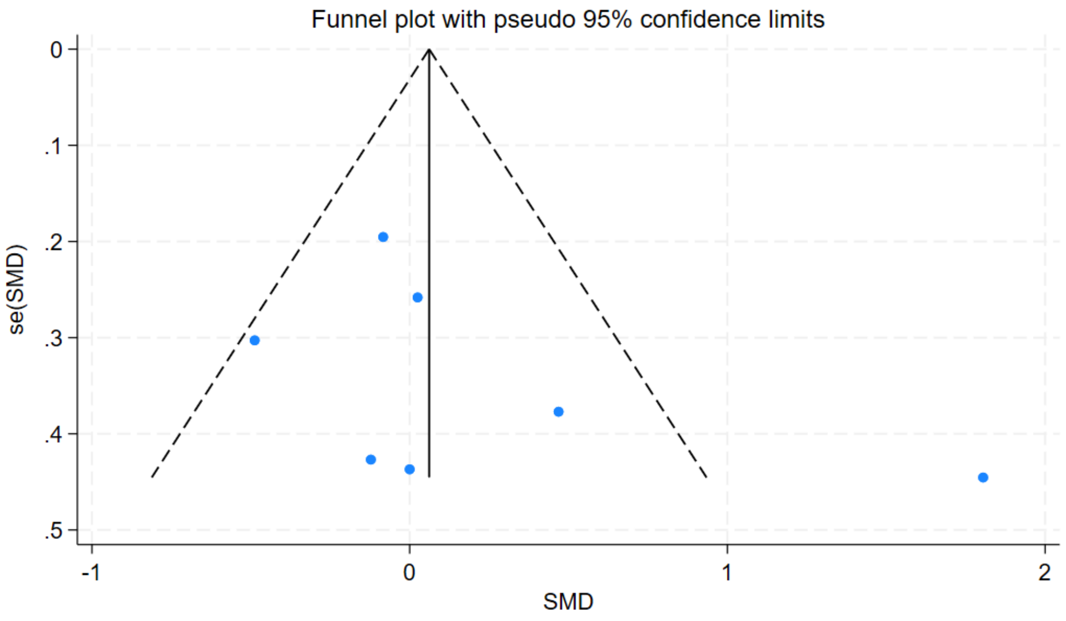

Fig.S4 Publication bias of ALB.

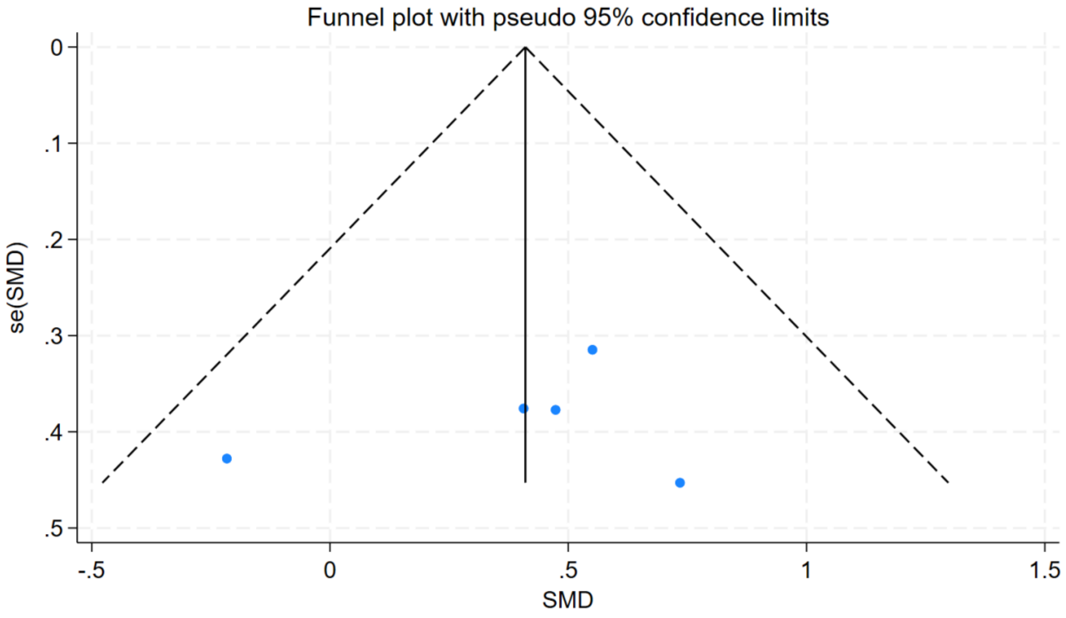

Fig.S5 Publication bias of BUN.

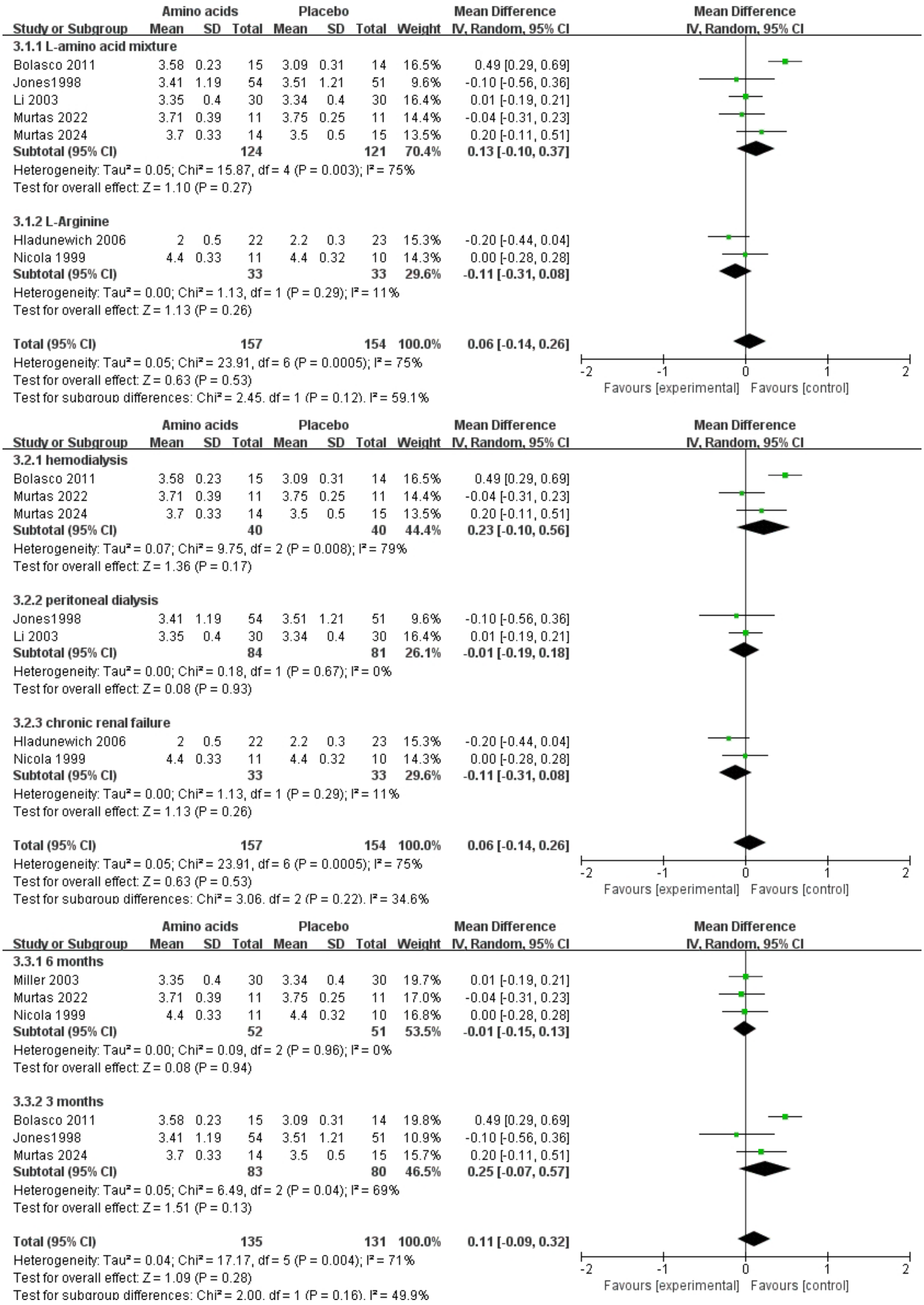

Fig.S6 Subgroup analysis of ALB by AA formulation type, RI subtype, and intervention duration.
